# *Plasmodium falciparum Maf1* Confers Survival upon Amino Acid Starvation

**DOI:** 10.1128/mBio.02317-16

**Published:** 2017-03-28

**Authors:** Kyle Jarrod McLean, Marcelo Jacobs-Lorena

**Affiliations:** Department of Molecular Microbiology and Immunology, Johns Hopkins Bloomberg School of Public Health, Johns Hopkins Malaria Research Institute, Baltimore, Maryland, USA; NIAID/NIH

**Keywords:** *Plasmodium*, TOR pathway, malaria

## Abstract

The target of rapamycin complex 1 (*TORC1*) pathway is a highly conserved signaling pathway across eukaryotes that integrates nutrient and stress signals to regulate the cellular growth rate and the transition into and maintenance of dormancy. The majority of the pathway’s components, including the central *TOR* kinase, have been lost in the apicomplexan lineage, and it is unknown how these organisms detect and respond to nutrient starvation in its absence. *Plasmodium falciparum* encodes a putative ortholog of the RNA polymerase (Pol) III repressor *Maf1*, which has been demonstrated to modulate Pol III transcription in a TOR-dependent manner in a number of organisms. Here, we investigate the role of *P. falciparum Maf1* (*PfMaf1*) in regulating RNA Pol III expression under conditions of nutrient starvation and other stresses. Using a transposon insertion mutant with an altered *Maf1* expression profile, we demonstrated that proper *Maf1* expression is necessary for survival of the dormancy-like state induced by prolonged amino acid starvation and is needed for full recovery from other stresses that slow or stall the parasite cell cycle. This *Maf1* mutant is defective in the downregulation of pre-tRNA synthesis under nutrient-limiting conditions, indicating that the function of *Maf1* as a stress-responsive regulator of structural RNA transcription is conserved in *P. falciparum*. Recent work has demonstrated that parasites carrying artemisinin-resistant *K13* alleles display an enhanced ability to recover from drug-induced growth retardation. We show that one such artemisinin-resistant line displays greater regulation of pre-tRNA expression and higher survival upon prolonged amino acid starvation, suggesting that overlapping, *Pf*Maf1-associated pathways may regulate growth recovery from both artemisinin treatment and amino acid starvation.

## IMPORTANCE

Eukaryote organisms sense changes in their environment and integrate this information through signaling pathways to activate response programs to ensure survival. The *TOR* pathway is a well-studied signaling pathway found throughout eukaryotes that is known to integrate a variety of signals to regulate organismal growth in response to starvation and other stresses. The human malaria parasite *Plasmodium falciparum* appears to have lost the *TOR* pathway over the course of evolution, and it is unclear how the parasite modulates its growth in response to starvation and drug treatment. Here, we show that Maf1, a protein regulated by *TOR* in other eukaryotes, plays an important role in maintaining the parasite’s viability in the face of starvation and other forms of stress. This suggests that *PfMaf1* is a component of a yet-to-be-described nutrient and stress response pathway.

## INTRODUCTION

Efficacy of the frontline antimalarial artemisinin is decreasing throughout Cambodia and other parts of Southeast Asia (1). The drug resistance phenotype presents as persistence, or delayed clearance, of ring-stage parasites in the peripheral blood after drug treatment for hours after susceptible parasites would have been cleared (2). Early theories derived from *in vitro* studies of susceptible parasites treated with drug proposed the so-called “sleeping beauty” hypothesis: ring-stage resistant parasites enter a state of dormancy, arresting the cell cycle and decreasing metabolic activity to limit damage, and then resume growth after drug concentrations have decayed below effective levels (3–8). However, more-recent work using field isolates known to carry mutations conferring the delayed clearance phenotype has cast doubt on the model of full cell cycle arrest and a transition to dormancy. Instead, studies have suggested that artemisinin derivatives induce a transient slowing of the cell cycle (9) and that parasites carrying resistance-conferring mutations are able to survive this retardation and resume growth, while susceptible parasites are not able to do so (10). The mechanisms by which the drug transiently slows the cell cycle and how the resistant alleles allow recovery remain unclear.

The yeast *Saccharomyces cerevisiae* has long been a model for the study of induction and maintenance of dormancy. When nutrients are depleted in the stationary phase, cells appear to cease progress through the mitotic cycle and enter a state of low metabolic activity and protein synthesis. This state serves a protective function, as yeast deprived of carbohydrates can remain viable and recover after more than 100 days of starvation (11).

Asexual stages of *Plasmodium falciparum* enter a state reminiscent of the yeast stationary phase upon nutrient limitation *in vitro*. When deprived of extracellular isoleucine (Ile), the only amino acid that cannot be obtained from the digestion of hemoglobin, the parasite dramatically slows its cell cycle and exhibits decreased protein synthesis and metabolic activity (12). The parasite can remain in this state for several days and resume normal growth upon isoleucine resupplementation with little loss in viability (12). To date, no parasite genes or pathways have been implicated in the attainment or maintenance of this dormancy-like state.

A likely candidate to govern these responses is the target of rapamycin complex 1 (*TORC1*) pathway, as it is known to integrate a range of positive and negative growth signals, most notably the presence of amino acids, to drive or inhibit cellular growth (13). The *TORC1* pathway is highly conserved throughout eukaryotes and was likely a central signaling hub in the last eukaryote common ancestor (14, 15). In mammalian cells in culture, inhibition of *mTOR* with the drug sirolimus induces G_1_ arrest in certain cell types but only slows cell cycle progression in others (16). In yeast, complete chemical or genetic inhibition of *TOR* drives cells into dormancy or quiescence (17), while partial inhibition slows cell cycle progression (18). A genome-wide deletion screen in yeast found *TORC1* pathway components to be the top hits for the ability to maintain viability in stationary phase during prolonged starvation (18).

**FIG 1:**
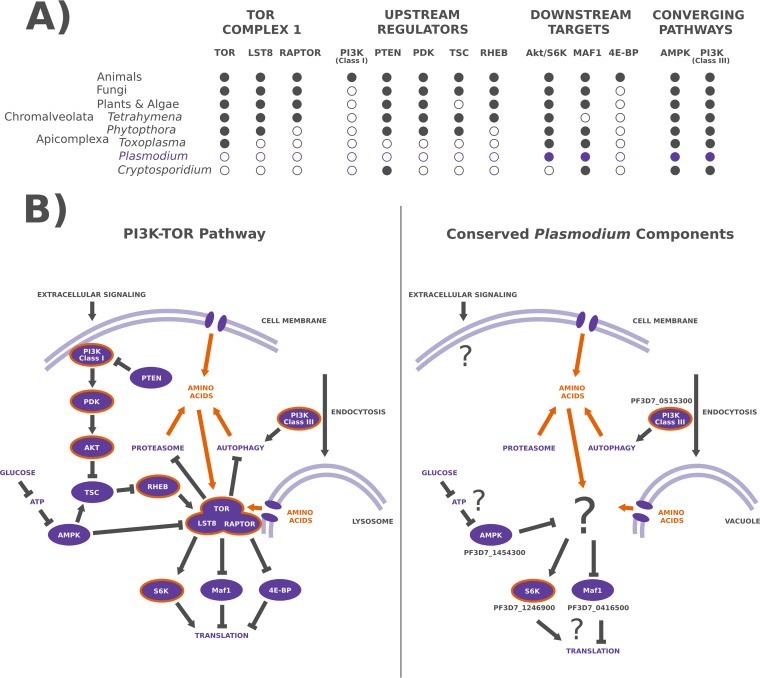
Few components of the TORC1 pathway remain in the *P. falciparum* genome. (A) A simplified representation of the major *TOR* complex 1 (*TORC1*) components, regulators, targets, and converging pathways across several eukaryote lineages. Solid circles (●) indicate the presence of the gene coding for the component in representative members of the lineage, and hollow circles (O) indicate the absence of the corresponding component in the lineage. (B) An illustration of the generalized animal *PI3K-TORC1* signaling cascade used to regulate cellular growth in the presence of amino acids and other growth factors (left) and a projection of this pathway in *Plasmodium* spp. based on the conserved components (right). *Plasmodium* spp. lack a class I *PI3K* enzyme and the other components (*PTEN*, *PDK*, an *Akt* homolog containing a PH domain) typically associated with this signaling cascade. *Plasmodium* spp. do encode a class III *PI3K* enzyme (PF3D7_0515300) whose ortholog has been implicated in *TORC1* signaling in human cells, as well as a PH domain lacking *PKB* family kinase (PF3D7_1246900) resembling human *S6K*. The genomes of *Plasmodium* parasites also encode an apparent ortholog of the *TORC1*-dependent RNA polymerase III regulator *Maf1* (PF3D7_0416500).

The majority of the most familiar components of the *TORC1* pathway, notably the *TOR* kinase itself, have been lost through genomic reduction in the evolution of the apicomplexan lineage (Fig. 1) (14, 15). While *P. falciparum* encodes none of the core *TORC1* components, several highly conserved genes that have been peripherally associated with various branches of *TORC1* remain. Intriguingly, two such components were recently associated with artemisinin resistance in parasites carrying K13 resistance alleles. Increased levels and activity of both the *P. falciparum* phosphatidylinositol 3-kinase gene (*PfPI3K*) (PF3D7_0515300) and *PfPKB* (also known as *PfAkt*) (PF3D7_1246900) were shown to confer increased survival of dihydroartemisinin (DHA) treatment *in vitro* (19), suggesting that these genes may actively regulate the slow growth and recovery program required to survive artemisinin treatment in the absence of *TORC1*.

A more recently appreciated factor associated with the *TORC1* pathway is the RNA polymerase (Pol) III regulator *Maf1*. Under nutrient-replete conditions, *TOR* signaling maintains *Maf1* in a phosphorylated and inactive state (20–23). Under starvation conditions, inhibition of *TORC1* leads to *Maf1* dephosphorylation, allowing it to bind to the RNA Pol III holoenzyme inhibiting Pol III-dependent transcription of tRNAs, the 5S RNA, and other structural RNAs (24, 25). Yeast *Maf1* null mutants die under conditions of nutrient limitation or when other factors inhibit *TORC1*, due to inability to regulate Pol III transcription (22, 26–28). In yeast, *Maf1* is one of the most important genes for maintaining viability during long-term starvation in stationary phase (18, 29). A putative *Maf1* ortholog appears to be conserved in the *Plasmodium* genus.

Here, we show that the *P. falciparum* gene is a functional ortholog of yeast *Maf1*. A transposon insertion line of *P. falciparum* with defective *Maf1* expression is unable to regulate Pol III activity or to maintain viability during the dormancy-like state induced by isoleucine starvation. This mutant displays additional growth and recovery defects for a range of growth-inhibiting forms of stress. Furthermore, an artemisinin-resistant isolate displays more effective Pol III regulation and increased survival upon amino acid starvation, suggesting that *PfMaf1* remains a downstream effector of growth regulation pathways in *Plasmodium falciparum* despite its loss of *TORC1*.

## RESULTS

### PF3D7_0416500 is a functional ortholog of *Maf1*.

The *Plasmodium falciparum* gene PF3D7_0416500 encodes a putative ortholog of *Maf1*. As with many *P. falciparum* proteins, the *P. falciparum* ortholog contains a large, asparagine-rich low-complexity region near the N terminus accounting for 200 (51%) of the 389 amino acids of the protein. This low-complexity region is not conserved in other *Plasmodium* species, and when it is not considered, the *P. falciparum* sequence aligns well with the region of the human ortholog used in determining the crystal structure (PDB accession number 3NR5) (25) and with the corresponding regions of the *S. cerevisiae* and *Schizosaccharomyces pombe* proteins (see Fig. S1 in the supplemental material).

10.1128/mBio.02317-16.1 FIG S1  The sequence of the *Plasmodium falciparum* core region of *Maf1* is conserved. Results of a Clustal Omega alignment of core *Maf1* protein sequences from human (3NR5), *Saccharomyces cerevisiae* (Sc), *Schizosaccharomyces pombe* (Sp), and *Plasmodium falciparum* (Pf) are shown. The *Maf1* protein contains two unstructured regions that display negligible sequence conservation between species. These regions were removed for the production of the *Homo sapiens* crystal structure (PDB accession number 3NR5). The corresponding regions of the other species were omitted for clarity. The sequences used for alignment were as follows: for *H. sapiens* (NC_000008.11), positions 1 to 35 and 83 to 210; for *S. cerevisiae* (YDR005C), 1 to 35 and 225 to 342; for *S. pombe* (SPAC31G5.12c), 1 to 35 and 78 to 197; and for *P. falciparum* (PF3D7_0416500), 1 to 36 and 224 to 349. Download FIG S1, TIF file, 0.6 MB. Copyright © 2017 McLean and Jacobs-Lorena. 2017 McLean and Jacobs-Lorena This content is distributed under the terms of the Creative Commons Attribution 4.0 International license.

**FIG 2:**
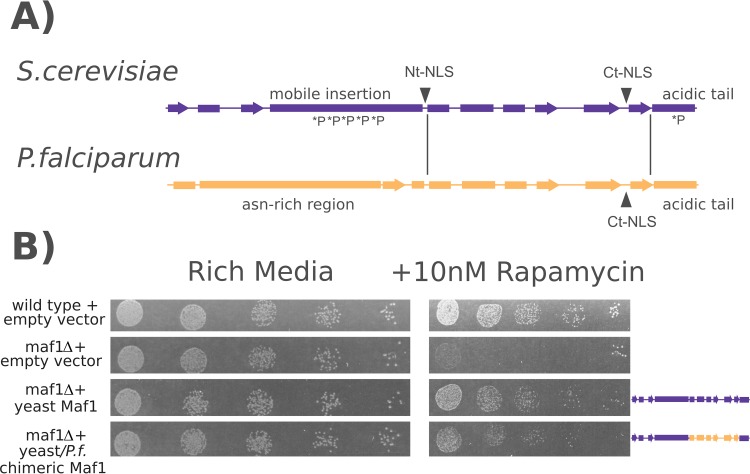
Functional complementation of Maf1-knockout yeast cells with a chimeric *P. falciparum* Maf1. (A) Schematics showing the key structural features of the yeast and *Plasmodium Maf1* orthologs (Nt, N terminus; Ct, C terminus; NLS, nuclear localization signal). Vertical lines indicate the homologous region exchanged to generate the chimera for complementation. *P, known site of phosphorylation in the yeast protein. (B) Fivefold serial dilutions of from ~5,000 to ~8 yeast cells transformed with the indicated complementation vectors were plated on normal rich media or on rich media supplemented with 10 nM sirolimus, which is lethal to *Maf1* knockout yeast cells. Images are representative of one of three biological replicates.

In yeast, *Maf1* knockout cells grown on a nonfermentable carbon source or treated with the TORC1 inhibitor sirolimus display severe growth defects and death (27). To test whether the *P. falciparum* gene is a true functional ortholog of *Maf1*, we attempted to complement this phenotype in yeast. *Maf1* is regulated by phosphorylation in both yeast and human cells; however, the regions in which the phosphorylation occurs are the least conserved. In yeast, phosphorylation has been described in two regions, the “mobile insertion” near the N terminus (so named for its sensitivity to limited proteolysis in recombinant proteins) and the “acidic tail” at the C terminus (the C termini of all *Maf1* orthologs, including *P. falciparum*, have high levels of aspartate and glutamate content but otherwise show little sequence identity) (25). In order to account for these phosphoregulatory regions, we generated a chimeric *Maf1* sequence consisting of the yeast N terminus and mobile insertion, the *PfMaf1* central core, and the yeast acidic tail (Fig. 2A). While this chimera may appear to contain a small portion of the *P. falciparum* sequence, the region used spans 67% of the core region of the human ortholog used in producing the crystal structure (25).

Under conditions that included the use of medium containing 10 nM sirolimus, wild-type (WT) yeast cells were able to grow but *Maf1*-knockout cells were not (Fig. 2B). When either the full-length yeast protein or the yeast-*P. falciparum* chimera was expressed from an episomal plasmid, partial levels of growth were equally restored, indicating that the core region of the *P. falciparum* ortholog is capable of functionally complementing yeast knockout cells.

### The PB-11 parasite clone carries a transposon insertion upstream of the *Maf1* open reading frame (ORF).

We attempted to disrupt the *Maf1* ortholog PF3D7_0416500 with several different plasmids for single- and double-crossover recombination, including multiple attempts using clustered regularly interspaced short palindromic repeat (CRISPR)–CRISPR-associated protein-9 nuclease (CRISPR-Cas9)/guide RNA. None of our efforts was successful, raising the possibility that this gene is essential for intraerythrocytic growth. We were not successful in integrating any C-terminal epitope tags or destabilization domains (no 3′ integration was ever detected by PCR in transfected parasites), suggesting that an unmodified C terminus may also be necessary for proper function.

Surprisingly, we were also unable to express ectopic copies of *Maf1* in wild-type parasites, using either episomal plasmids or chromosomal integration via the *attB*/*attP* system (30). We attempted the use of both N-terminal and C-terminal tags, as well as both strong (eEF1α) (31) and weak (mRPL2) (32) promoters, but no stable transfectants could be obtained. Unrelated control plasmids routinely produced stable transfectants. Only a single ectopic expression construct produced stable parasites, and it consisted of the *P. falciparum Maf1* coding sequence fused to a “*DD24*” N-terminal FKBP12 destabilization domain (33) expressed from the weak *mRPL2* promoter. The resulting line expressed the *Maf1* transgene well below endogenous levels and was unresponsive to protein stabilization with the ligand *Shield*, limiting its utility in any further analyses (Fig. S2). These failed efforts suggest that *Maf1* may be toxic to cells if expressed at the wrong time and/or level.

10.1128/mBio.02317-16.2 FIG S2  Ectopic expression of *PfMaf1* is attainable only using a construct designed to attenuate protein expression. (A) A schematic of the transgene used for transfection. A 3× HA tag followed by a *DD24* destabilization domain was fused to the N terminus of the *Maf1* coding sequence and driven by the weak mitochondrial ribosomal protein 2 (mRPL2) promoter. (B) A Western blot of schizonts of untransfected NF54 parasites or Dd2 stably transfected with the *DD24*-*Maf1* expression plasmid probed with anti-*PbMaf1* antisera. The 3× HA and *DD24* tags are expected to add 15 kDa to the *PfMaf1* molecular mass. Increasing concentrations of the *Shield* ligand are expected to increase the stability and abundance of the *DD24-Maf1* protein but in this case had no effect. Download FIG S2, TIF file, 1.8 MB. Copyright © 2017 McLean and Jacobs-Lorena. 2017 McLean and Jacobs-Lorena This content is distributed under the terms of the Creative Commons Attribution 4.0 International license.

**FIG 3:**
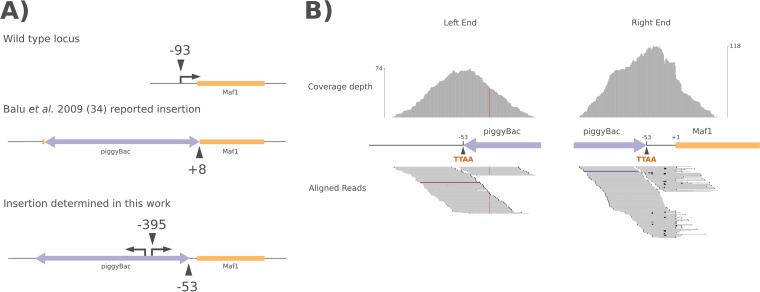
The PB-11 mutant carries a *piggyBac* insertion in the 5′UTR of the *Maf1* gene. (A) Schematics of the *Maf1* genomic locus and *piggyBac* insertion in wild-type and mutant parasites. Numbers below the chromosomal line indicate the reported and verified transposon insertion positions relative to the *Maf1* start codon. Right-angled arrows and numbers above the chromosome line indicate the transcription start sites in wild-type (−93 nt) and PB-11 mutant (−395 nt) cells as determined by 5′-RACE. The *Maf1* transcript in the PB-11 mutant arises from within the *piggyBac* transposon and is likely due to bidirectional promoter activity of the *calmodulin* promoter fragment used for drug selection in the transposon. (B) The upstream insertion site was confirmed by whole-genome sequencing (top). Coverage depth plots indicate the total number of reads mapping to each base pair around the two termini of the *piggyBac* insertion at the −53 TTAA site upstream of the *Maf1* start codon. The maximum read coverage for each end of the transposon is indicated by the axes on the left and right (bottom). Raw reads aligned to the termini of the transposon surrounding the −53 insertion site. Each read is 100 bp in length. Colored spots indicate base mismatches within a given read and the genomic sequence.

In their efforts to generate a *piggyBac* mutant collection, Balu et al. generated a *Maf1* transposon insertion mutant in the NF54 parasite background (34). In this clone, identified as PB-11, Balu et al. reported the *piggyBac* transposon to be inserted at the TTAA sequence ending at position +8 of the ORF, which should disrupt the coding sequence to generate a genetic knockout (Fig. 3A).

We obtained this mutant and discovered that the insertion site had been incorrectly assigned. Instead of the +8 position of the ORF, the insertion is at a TTAA sequence 53 bp upstream of the start codon. Whole-genome sequencing of the PB-11 line (Illumina HiSeq paired-end sequencing, 73× coverage) demonstrated extensive read coverage for the −53 insertion position and no support for a +8 insertion site (Fig. 3B). To rule out the possibility of additional, unknown *piggyBac* insertions in the genome, we aligned one file of the paired reads to the transposon sequence and the pair file to the genome (and vice versa) to see where each transposon-aligning paired read mapped (35). Paired ends were found to align only to the *Maf1* locus or to regions of the genome used as regulatory sequences within the transposon itself (such as the *calmodulin* promoter or the *HrpII* 3′ untranscribed region [3′UTR]) (Table S1). Additionally, we analyzed the PB-11 genome for single nucleotide polymorphisms (SNPs) and indels within all annotated open reading frames in the genome but found none of significance compared to the parental line or a sister *piggyBac* insertion mutant sequenced previously (35) (Table S2). The latter results support the notion that any phenotype observed in the PB-11 mutant line is a consequence of the transposon insertion.

10.1128/mBio.02317-16.7 TABLE S1  Verifying the PB-11 transposon insertion site using paired-end reads. The PB-11 genome was sequenced using paired-end sequencing. This process generates paired reads, where each pair represents opposite ends of the genomic fragment generated during library preparation. A genomic fragment that spans the transposon integration site should contain one read aligning to the *piggyBac* transposon sequence and a paired read aligning to the genome near the site of integration. All reads were aligned independently to the *piggyBac* transposon and the *Plasmodium falciparum* reference genome. For any read that aligned to the transposon, the genomic location of its partner read was determined. The paired reads of all transposon mapping reads aligned to only five genomic “windows.” Many paired reads aligned to the *Maf1* locus, confirming the insertion site of the transposon. The remaining aligned reads mapped to the calmodulin or hrpII (or the nearly identical hrpIII) locus. Regions from these loci were used for the promoter and terminator of the drug selection cassette in the transposon. Alignment of reads to these loci most likely reflects genomic fragments contained entirely within the transposon itself instead of additional integration events at these loci. Download TABLE S1, TIF file, 0.4 MB. Copyright © 2017 McLean and Jacobs-Lorena. 2017 McLean and Jacobs-Lorena This content is distributed under the terms of the Creative Commons Attribution 4.0 International license.

10.1128/mBio.02317-16.8 TABLE S2  List of potential in-ORF variants in the PB-11 mutant identified by whole-genome sequencing. Whole-genome sequencing analysis of the PB-11 mutant identified 32 potential variants within open reading frames. Read alignments of each variant were visually inspected. All 32 variants could be explained as sequencing artifacts due to highly repetitive regions or mononucleotide tracts. Download TABLE S2, TIF file, 1.2 MB. Copyright © 2017 McLean and Jacobs-Lorena. 2017 McLean and Jacobs-Lorena This content is distributed under the terms of the Creative Commons Attribution 4.0 International license.

### The PB-11 mutant displays an altered *Maf1* expression profile.

**FIG 4:**
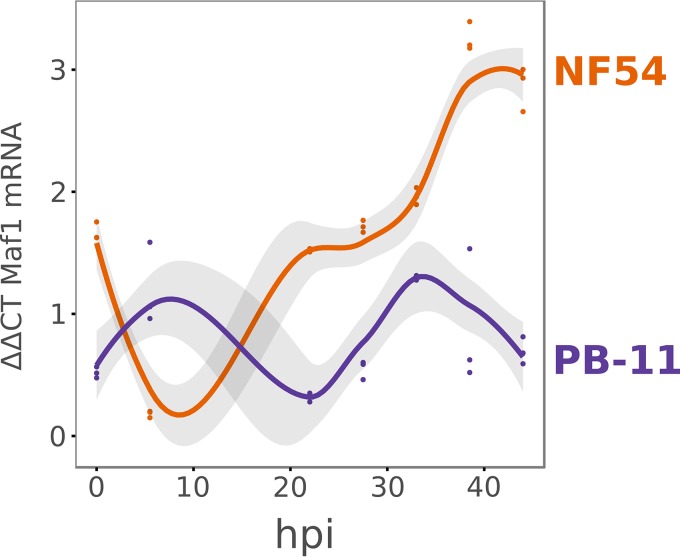
The PB-11 mutant displays an abnormal *Maf1* mRNA expression profile. An analysis of the time course of *Maf1* mRNA expression was performed using qRT-PCR at seven time points across the intraerythrocytic cycle and synchronous wild-type (NF54) and mutant (PB-11) parasites. *Maf1* expression was quantified relative to that of the seryl-tRNA ligase transcript (PF3D7_0717700). Points represent individual biological replicates (three in total), and curves represent LOESS smoothed models fitted to the data, with the 95% confidence interval indicated by shading. ΔΔCT, threshold cycle method; hpi, hour post-red blood cell (RBC) invasion.

The −53 insertion in the PB-11 line does not ablate expression of *Maf1*. The *Maf1* transcript is detectable in PB-11 mutants throughout the asexual life cycle but does not match the wild-type expression pattern (Fig. 4) (Fig. S3). In later stages of the cell cycle, when wild-type *Maf1* mRNA abundance dramatically increases, expression of mutant *Maf1* stays at early-cycle levels.

10.1128/mBio.02317-16.3 FIG S3  Comparison of *Maf1* mRNA expression at individual time points across the intraerythrocytic cycle. The same data as described for Fig. 4 are represented as mean relative expression bar plots. *P* values represent results of *t* tests for differences in expression between wild-type (WT) and mutant (PB-11) parasites at each time point across three biological replicates. Error bars represent standard deviations. Download FIG S3, TIF file, 0.5 MB. Copyright © 2017 McLean and Jacobs-Lorena. 2017 McLean and Jacobs-Lorena This content is distributed under the terms of the Creative Commons Attribution 4.0 International license.

The insertion of an ~3 kb transposon at 53 bp upstream of the start codon would normally be expected to disrupt the activity of the native promoter sequence. Therefore, we performed 5′ rapid amplification of cDNA ends (5′-RACE) on *Maf1* mRNA from both wild-type and PB-11 mutant parasites. In the wild-type cells, we mapped the transcription start site to 93 bp upstream of the *Maf1* start codon, meaning that *piggyBac* inserted into the 5′UTR in the PB-11 mutant (Fig. 3A). In PB-11 cells, the *Maf1* transcription start site mapped to 395 bp upstream of the start codon, producing a chimeric 5′UTR consisting of 346 bp of the right arm of the *piggyBac* transposon (Fig. 3A). In constructing the transposon pXL-BacII-DHFR (pXL-BacII-dihydrofolate reductase), Balu et al. used the full intergenic region between the start codons of the gene encoding calmodulin (PF3D7_1434200) and its opposite gene (PF3D7_1434300) to drive expression of the human DHFR (hDHFR) selectable marker (36). It appears that this region has enough promoter activity to drive transcription of the chimeric *Maf1* mRNA in PB-11 in the direction opposite that of the hDHFR gene. This promoter has been previously reported to be bidirectional when used in plasmids (37).

Antisera raised against full-length *Plasmodium berghei Maf1* detected accumulation of *Maf1* protein in both wild-type and mutant cells. The *Maf1* product is a low-abundance protein and was largely undetectable during the first 24 h of the life cycle (Fig. S4A). By 27.5 h postinvasion (hpi), *Maf1* was detectable in wild-type parasites but was not yet detectable in PB-11 parasites (Fig. S4B). It was detectable, however, in the later hours of the cell cycle at near wild-type levels despite the much lower mRNA abundance. A recent global ribosome profiling study of the *Plasmodium falciparum* asexual cycle found *Maf1* to be translated at very low efficiency from rings through schizonts (38). Perhaps the chimeric 5′UTR generated by the *piggyBac* insertion removes the *cis*-acting factors in the native 5′UTR responsible for the observed low translational efficiency, allowing protein production at low mRNA levels, albeit with a notable temporal delay in expression. This endogenous translational control may explain why we were able to obtain stable transfected lines only when expressing *Maf1* from a construct designed to reduce protein production levels (Fig. S2).

10.1128/mBio.02317-16.4 FIG S4  The PB-11 insertion mutant displays delayed *Maf1* protein expression. (A) Western blot of *Maf1* expression in synchronous wild-type (NF54) and mutant (PB-11) parasites at 5.5-h intervals across the first 22 h of the intraerythrocytic cycle. No *Maf1* protein expression was detectable in either parasite line even after prolonged exposure. (B) Western blot of *Maf1* protein expression at 5.5-h intervals from 27.5 h through 44 h of the cell cycle. hpi, hour post-RBC invasion. Download FIG S4, TIF file, 0.6 MB. Copyright © 2017 McLean and Jacobs-Lorena. 2017 McLean and Jacobs-Lorena This content is distributed under the terms of the Creative Commons Attribution 4.0 International license.

The *piggyBac* transposon is well known for its ability to excise cleanly from its insertion site when remobilized (39). We attempted to use this property to restore the wild-type *Maf1* locus in the PB-11 mutant line by excising *piggyBac*. While we were able to obtain parasites stably expressing the *piggyBac* transposase, no excision of the insertion was detected. Similarly, we attempted to restore the native locus by “crossing out” the *piggyBac* insertion via *CRISPR-Cas9*-mediated homologous recombination. We attempted several different strategies with different guide RNAs; however, all failed to restore the wild-type locus.

### The PB-11 *Maf1* mutant fails to recover from a dormancy-like state.

*TORC1* is best understood as the central hub from which the progrowth signaling cascades are relayed in response to changes in intracellular levels of amino acids. In yeast, human cells, and *Drosophila* grown under normal conditions, *TORC1* is active and *Maf1* is inhibited (20, 27, 40). When these organisms are starved of amino acids (or *TORC1* is inhibited by sirolimus), *Maf1* is activated and shuts down Pol III-dependent transcription of tRNAs and other structural RNAs to conserve resources and globally dampen translation. If this safety mechanism is prevented by deletion or silencing of *Maf1*, death or severe growth defects result upon amino acid starvation.

Over the course of the asexual cell cycle, *Plasmodium falciparum* resides within its host erythrocyte and imports and digests hemoglobin as its primary source of amino acids. Human hemoglobin lacks isoleucine, and since the parasite does not have the capacity to synthesize it itself, it must import isoleucine from the extracellular environment (i.e., serum *in vivo* or culture medium *in vitro*). Isoleucine is the only amino acid needed in the culture medium to sustain growth of *P. falciparum in vitro* (41).

Unlike the results seen with nutrients such as glucose, whose removal led to rapid death, Babbit et al. found that *P. falciparum* parasites appear to retard growth and enter a dormancy-like state of low metabolic activity when starved of isoleucine (12). Upon resupplementation, parasite growth resumed seemingly unaffected. Parasites could remain in the absence of isoleucine for upwards of several days and sustain only a minor loss in viability. This is reminiscent of the stationary-phase dormancy observed in yeast, a process for which *Maf1* is necessary to maintain viability during prolonged starvation (18, 29).

Babbit et al. were unable to identify genetic factors responsible for this phenotype. Neither deletion of the *P. falciparum* homolog of the *GCN2* uncharged tRNA sensor kinase nor the expression of a nonphosphorylatable form of its target, initiation factor 2α (IF2α), had an effect on survival after isoleucine starvation (12). Given the role that *Maf1* plays in the amino acid starvation response in other organisms, we investigated how the *Maf1* PB-11 mutant responded to isoleucine withdrawal.

**FIG 5:**
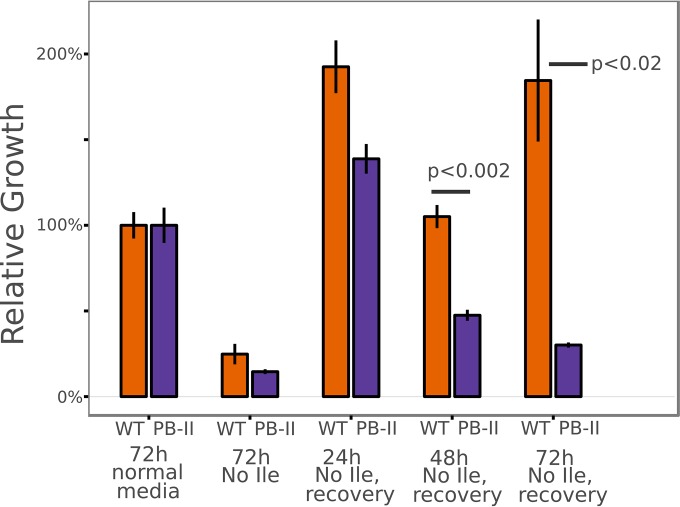
*Maf1* mutant parasites cannot recover from a prolonged dormancy-like state induced by isoleucine starvation. Synchronous young ring-stage parasites (approximately 4 h postinvasion) were washed repeatedly and transferred to medium lacking isoleucine (Ile) for the indicated times. Recovery data denote transfer back to normal culture medium (containing isoleucine) for 72 h of growth. Parasitemia was quantified by flow cytometry. Growth was measured relative to the final level of parasitemia of a control culture incubated in normal culture medium for 72 h. *P* values were calculated using *t* tests of three biological replicates.

Tightly synchronized young ring-stage wild-type and PB-11 mutant parasites were cultured for 72 h in either normal culture medium or medium lacking isoleucine. As previously reported, parasites in the isoleucine-deficient medium stayed at roughly their starting levels of parasitemia whereas those in normal medium grew through ~1.5 cell cycles. No detectable difference between wild-type and PB-11 parasites was observed over this period (Fig. 5).

We next tested whether the parasites remained viable over the duration of starvation. Young ring-stage parasites of both wild-type and PB-11 parasites incubated in medium lacking isoleucine for 24 h recovered when returned to normal culture medium for a 72-h recovery period (Fig. 5). However, when the starvation period was increased to 48 h, the PB-11 *Maf1* mutant line displayed a notable decrease in recovery, whereas wild-type parasites displayed growth levels similar to those seen with parasites cultured for 72 h in normal medium. With a 72-h starvation, PB-11 mutants displayed no recovery, while wild-type parasites recovered robustly.

**FIG 6:**
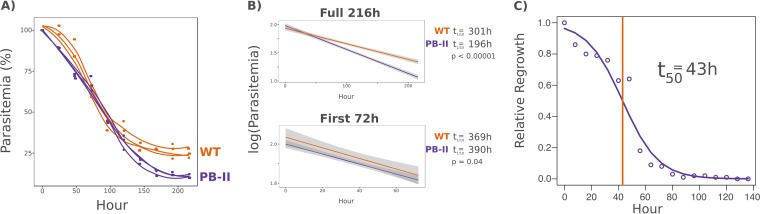
The PB-11 mutant remains viable but loses the ability to recover within the first 72 h of starvation. (A) Parasitemia of *Maf1* mutant parasites decreases more rapidly during prolonged isoleucine starvation. Synchronous young ring-stage NF54 (WT) and *Maf1* insertion mutant (PB-11) parasites were washed repeatedly and transferred to medium lacking isoleucine. Samples of each parasite line were taken at 8-h intervals over the course of a 9-day (216-h) period, and parasitemia was determined by flow cytometry. Lines represent LOESS curves fitted to the data for each of three biological replicates. (B) *Maf1* mutant parasites display minimal differences in death rate over the first 72 h of starvation. Regression models were fitted to the 216-h isoleucine starvation data to determine the rate of death of NF54 (WT) and *Maf1* mutant (PB-11) parasites over the full 216 h or for only the first 72 h of the same data set (t_1/10_ = time required to reach 1/10 the starting level of parasitemia). (C) Maf1 mutant parasites lose the ability to recover after (on average) 43 h of isoleucine starvation. Synchronous young ring-stage PB-11 parasites were washed and transferred to medium lacking isoleucine. Every 3 h, samples were transferred to normal medium for a 72-h recovery period. The final level of parasitemia after recovery was quantified by flow cytometry. A logistic regression fitted to the data shows that each hour of starvation decreases the parasitemia level to 93% of the level seen the previous hour (β_o_ = −0.076, *P* < 2.00 × 10^−16^). The logistic model fitted to the data predicts that the time point corresponding to 43 h of starvation is the point at which 50% of the PB-11 parasites are able to recover and 50% are not (t_50_). Data shown are the results of three biological replicates.

Despite not being a full gene disruption, the alteration of *Maf1* expression in the PB-11 mutant line appears to prevent the parasites from recovering from prolonged amino acid starvation. To investigate further, we used flow cytometry to track the parasitemia of wild-type and PB-11 cultures every 24 h over 9 days (216 h) of starvation in isoleucine-deficient medium (Fig. 6A). Over the full course of the 9 days, the PB-11 parasitemia decreased at a much higher rate (time required to reach 1/10 the starting level of parasitemia [*t*_1/10_] for the WT, 301 h; *t*_1/10_ PB-11, 196 h) (Fig. 6B). Over the first 72 h, however, the death rates of the two lines were nearly identical (*t*_1/10_ WT, 390 h; *t*_1/10_ PB-11, 369 h) (Fig. 6B). As a verification, aliquots of each replicate sample were transferred to normal medium for 3 days of recovery at the 72-h, 144-h, and 216-h points of starvation (Fig. S5). PB-11 displayed no regrowth at any time point, whereas wild-type parasites remained viable and recovered after 9 days.

10.1128/mBio.02317-16.5 FIG S5  Wild-type parasites remain capable of recovery over the full duration of 216 h of starvation. The data presented represent a supplement to Fig. 6A. Samples of wild-type (NF54) and *Maf1* mutant (PB-11) parasites taken after 72 h, 144 h, and 216 h of isoleucine starvation were transferred to normal culture medium for 72 h of recovery. The final parasitemia levels were quantified by flow cytometry, and data are expressed as fold growth relative to the level of parasitemia at the time of transfer. *P* values represent results of *t* tests across three biological replicates. Error bars represent standard deviations. Download FIG S5, TIF file, 0.6 MB. Copyright © 2017 McLean and Jacobs-Lorena. 2017 McLean and Jacobs-Lorena This content is distributed under the terms of the Creative Commons Attribution 4.0 International license.

Babbitt et al. reported that isoleucine starvation in young rings causes cells to progress through the early stages of the cell cycle at approximately 40% the normal rate of growth prior to arrest in the mid-trophozoite stage (12). If PB-11 *Maf1* mutant cells can fully recover from a 24-h isoleucine starvation but not at all from a 72-h starvation, this suggests that a specific point in the cell cycle is crossed between those two time points at which the ability to recover is irreversibly compromised, presumably due to altered *Maf1* expression.

To map this point, we initiated a culture of early-ring PB-11 parasites in isoleucine-deficient medium for a 144-h incubation (Fig. 6C). Every 8 h, an aliquot of the culture was removed and resuspended in normal medium for a 72-h recovery period. A logistic regression was performed using the relative proportion of parasites recovering at each time point. The results showed a loss of the ability to recover as the duration of the starvation period increased. According to the regression fitted to the data, the point of 50% recovery occurred at 43.1 h of starvation. Given that the cell cycle advances at approximately 40% the normal pace during starvation (12), this corresponds to some time point after 17 h postinvasion. This coincides with the time point at which differences in *Maf1* mRNA abundance were most pronounced (Fig. 4) and with the time point just prior to that at which differences in *Maf1* protein expression were most pronounced (Fig. S4).

### PB-11 displays growth and recovery defects when exposed to multiple growth-retarding stressors.

In yeast, *Maf1* arrests Pol III transcription in response to a broad array of biotic and abiotic stressors in addition to starvation (26). Since *TORC1* and, by extension, *Maf1* typically regulate progression and arrest of the cell cycle, we investigated whether stressors that stall or retard the cell cycle, in addition to isoleucine starvation, also cause growth or recovery defects for the PB-11 *Maf1* mutant.

**FIG 7:**
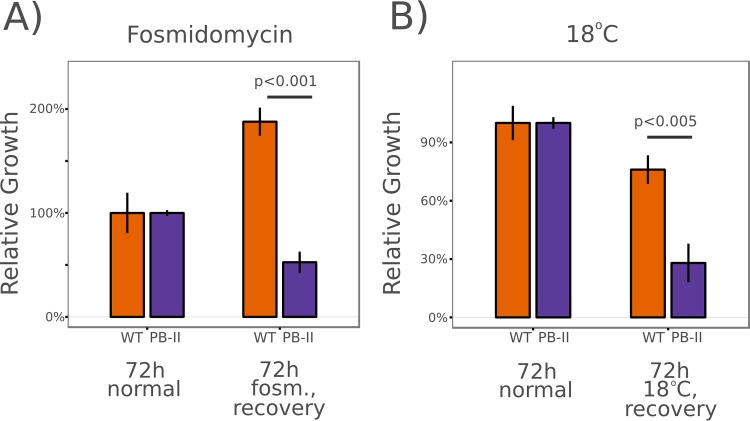
PB-11 *Maf1* mutant parasites display defects in recovery from fosmidomycin exposure and low-temperature treatment. (A) Young ring-stage NF54 (WT) and *Maf1* mutant (PB-11) parasites were incubated in the presence of 5 μM fosmidomycin (fosm.) for 72 h and were then “recovered” by incubation for a further 72 h in the presence of 5 μM fosmidomycin and 5 μM geranylgeraniol. Growth was measured relative to that of parasites incubated for 72 h in normal medium treated with both fosmidomycin and geranylgeraniol. (B) Young ring-stage parasites were incubated at 18°C for 72 h and were recovered by 72 h of growth at 37°C. Growth was measured relative to that of parasites incubated for 72 h at 37°C. *P* values were calculated using *t* tests of three biological replicates.

The antibiotic fosmidomycin inhibits isoprenoid biosynthesis in the *Plasmodium* apicoplast. Howe et al. previously demonstrated that treatment of parasites with 5 μM fosmidomycin causes parasites to seemingly arrest in mid-schizogeny (42). Subsequent treatment of cultures with the downstream isoprenoid geranylgeraniol liberates the parasites from arrest. We arrested wild-type and PB-11 mutant parasites with 5 μM fosmidomycin for 72 h and then treated them with 5 μM geranylgeraniol for a 72-h regrowth period and compared the levels of recovery to those seen with controls treated with fosmidomycin and geranylgeraniol simultaneously for 72 h (Fig. 7A). The PB-11 *Maf1* mutants displayed a pronounced decrease in recovery relative to the wild type.

Ring-stage *Plasmodium falciparum* is capable of surviving prolonged exposure to temperatures well below 37°C (43). Presumably, the low temperature slows the cell cycle in a manner that may also require proper *Maf1* expression for survival. We incubated wild-type and PB-11 *Maf1* mutant parasites at 18°C for 72 h and then returned the cultures to 37°C for 72 h of recovery and compared the growth to that seen with controls that remained at 37°C for 72 h (Fig. 7B). Once again, the *Maf1* mutant parasite line displayed notably decreased recovery from low-temperature stress relative to the wild-type results.

**FIG 8:**
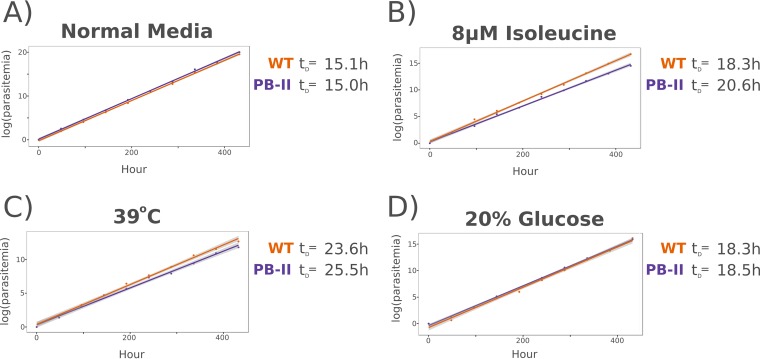
PB-11 *Maf1* parasites display a decreased growth rate under low-isoleucine conditions and at elevated temperatures. Growth curves were measured for NF54 (WT) and *Maf1* mutant (PB-11) parasites cultured in normal culture medium at 37°C (*P* < 0.0001) (A), 8 μM isoleucine (approximately 2% the concentration of normal medium) (*P* < 0.001) (B), normal culture medium at 39°C (*P* = 0.003) (C), and 20% (0.4 g/liter) of the glucose level of normal medium (2.0 g/liter) (*P* = 0.08) (D). *P* values represent results of tests of the parasite line (i.e., the wild type versus PB-11) as a factor in the regression analysis. t_D_, doubling time.

The growth rates of wild-type and *Maf1* mutant parasites were distinct, though similar, in culture in normal medium (Fig. 8A). However, cultured in a growth-limiting concentration of isoleucine (8 μM [approximately 2% of the normal RPMI 1640 concentration]), the doubling time of the PB-11 *Maf1* mutant was markedly longer (Fig. 8B). A similar phenomenon occurred when the parasites were cultured in normal medium at the stress-inducing temperature of 39°C (Fig. 8C). Interestingly, the growth rates of both the wild-type and PB-11 parasites were slowed by culture in medium containing 20% the normal RPMI 1640 glucose concentration (i.e., 0.4 g/liter), but the conditions used appeared to affect the two parasite clones equally (Fig. 8D). This may suggest that *Maf1* is not part of a universal stress response but is instead a component of a specific pathway that responds to certain growth-inhibiting and growth-retarding stressors but not to others.

### tRNA expression is dysregulated in the PB-11 *Maf1* mutant upon amino acid starvation.

*Maf1* is the only known regulator of RNA Pol III transcription. When conditions are unfavorable, *Maf1* is activated and binds to the Pol III holoenzyme, shutting down transcription of tRNAs, 5S rRNA, and a small number of other structural RNAs. This action is presumed to save cellular resources in times of stress and nutrient limitation and may also globally reduce translational output by limiting the amount of the available tRNA pool. Presumably, *Maf1* performs a similar function in *Plasmodium falciparum*.

**FIG 9:**
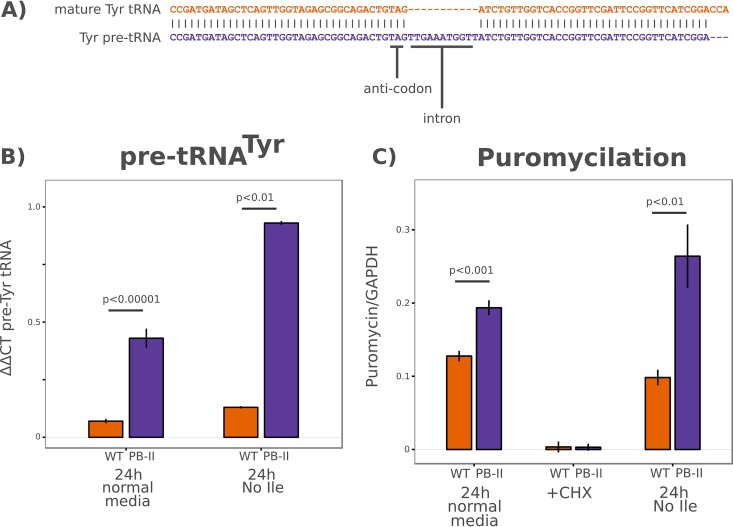
*Maf1* mutant parasites display elevated pre-tRNA expression and elevated global translation under normal and isoleucine starvation conditions. (A) An alignment of the genomic pre-tRNA^Tyr^ sequence and the mature tRNA^Tyr^ sequence reveals an 11-nucleotide intron adjacent the anticodon. (B) Stem-loop RT-qPCR profiling of pre-tRNA expression in NF54 (WT) and *Maf1* mutant (PB-11) parasites. Synchronous young ring-stage parasites were incubated in normal medium or medium lacking isoleucine for 24 h prior to RNA isolation. Pre-tRNA^Tyr^ expression was quantified relative to 5.8S rRNA levels. (C) ELISA of puromycin incorporation relative to GAPDH (glyceraldehyde-3-phosphate dehydrogenase) gene levels in wild-type and mutant parasites under conditions of the indicated treatments. Synchronous young ring-stage parasites were incubated in normal medium or medium lacking isoleucine for 24 h prior to the 1-h puromycin pulse and subsequent harvesting. Translational arrest by cycloheximide (CHX) treatment was used as a negative control. *P* values were calculated using *t* tests of three biological replicates.

The long half-life of mature tRNAs makes it difficult to quantify changes in their expression levels. Typically, pre-tRNAs, immature precursors containing 5′ and 3′ transcriptional leader sequences and introns, are quantified instead, as these species are rapidly processed, allowing the assessment of Pol III transcriptional activity. At present, no pre-tRNA sequences have been annotated in the *Plasmodium falciparum* genome. Nearly every eukaryote tyrosine tRNA gene contains an intron immediately 3′ of the anticodon, the processing of which is believed to be necessary for the pseudouridine modification found in the anticodon of all mature eukaryote tRNA^Tyr^ molecules (44). We reverse transcribed and cloned the mature *Plasmodium falciparum* tRNA^Tyr^ gene and found that an 11-nucleotide (nt) sequence adjacent to the anticodon is spliced out in the mature tRNA molecule (Fig. 9A). Using this short intron as a target sequence, we modified a stem-loop real-time quantitative PCR protocol optimized for microRNAs (miRNAs) (45, 46) to quantify pre-tRNA abundance using the similarly sized mature 5.8S rRNA (Pol I transcript) as a reference.

Under normal medium conditions, the PB-11 *Maf1* mutant parasites displayed higher steady-state expression of pre-tRNA (Fig. 9B), suggesting that *Maf1* may play a role in regulating tRNA expression in normal growth as well as under conditions of stress. After 24 h of isoleucine deprivation, the difference in pre-tRNA levels was even more pronounced (Fig. 9B), supporting the notion that the failure of *Maf1*-dependent regulation of tRNA expression contributes to this mutant’s inability to recover from amino acid starvation and other forms of stress.

It has been suggested that the increase in intracellular tRNA levels caused by genetic suppression of *Maf1* can cause a global increase in translation due to increased levels of the initiator tRNA (47). Regulating translation in times of stress is a fundamental component of most cellular stress responses because it allows cells to conserve resources, decrease the buildup of aggregates and unfolded proteins, and selectively translate specific subsets of mRNAs (48). If this process is counteracted by excessive translational initiation, the cell’s ability to survive stress may be seriously compromised.

To quantify global translation activity, we used a puromycylation assay (49). Briefly, the translation inhibitor puromycin incorporates into nascent peptide chains on actively translating ribosomes. A monoclonal antibody specific for puromycin can then be used to quantify levels of puromycin incorporation as a surrogate for global translational output. We pulsed parasite cultures for 1 h with puromycin prior to harvesting protein and then quantified puromycin incorporation by enzyme-linked immunosorbent assay (ELISA) using anti-*P. falciparum* glyceraldehyde-3-phosphate dehydrogenase genes (anti-*PfGAPDH*) as an internal control.

When cells were cultured in normal medium, the PB-11 *Maf1* mutant displayed moderately higher puromycin incorporation than the wild-type cells (Fig. 9C). However, after 24 h of isoleucine starvation, the PB-11 mutant displayed a dramatic increase in puromycin incorporation relative to the wild-type control. This inability to repress translation under conditions of amino acid starvation may further contribute to the inability of the mutant line to survive prolonged starvation.

### An artemisinin-resistant *K13* variant confers increased survival and greater control of pre-tRNA expression upon isoleucine starvation.

Artemisinin and its derivatives induce growth retardation in ring-stage parasites (9), and artemisinin-resistant isolates appear capable of recovering from this drug-induced growth inhibition whereas sensitive isolates cannot (10). We investigated whether this ability to overcome growth retardation translated to increased recovery from prolonged amino acid starvation and whether drug-resistant parasites displayed differential levels of regulation of Pol III transcription as a potential point of connection between the artemisinin resistance phenotype and *PfMaf1*.

**FIG 10:**
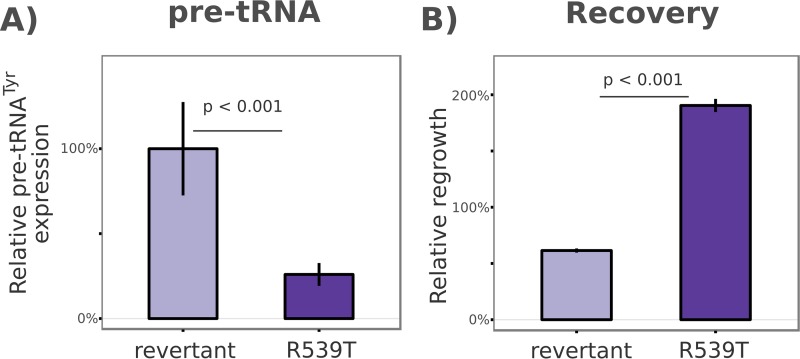
An artemisinin-resistant mutant displays increased pre-tRNA regulation and survival upon isoleucine starvation. (A) qPCR quantification of pre-tRNA^Tyr^ expression after 24 h of isoleucine starvation in an artemisinin-resistant field isolate (R539T) and a syngeneic clone converted to the artemisinin-sensitive *K13* allele (revertant). Data are represented as a percentage of the “wild-type” revertant expression level. (B) Sensitive (revertant) and artemisinin-resistant (R539T) parasites were starved of isoleucine for 72 h and then transferred to normal medium for 72 h of recovery. Growth was quantified by flow cytometry, and data are expressed as a percentage of the starting level of parasitemia of each line. The *P* values reflect *t* tests of three biological replicates.

Using an artemisinin-resistant field isolate originating from Battambang, Cambodia (*K13* allele: R539T), and a syngeneic line in which the *K13* was reverted to the sensitive allele (50), we cultured the parasites in the absence of isoleucine and evaluated their ability to regulate pre-tRNA expression and recover from starvation (Fig. 10A). After 24 h of isoleucine starvation, the R539T-resistant isolate displayed 27% the expression level of pre-tRNA seen with the syngeneic revertant line. Consistent with our observation in the PB-11 *Maf1* mutant, the ability to suppress pre-tRNA expression coincided with increased survival. After 72 h of isoleucine starvation, and a subsequent 72 h of recovery, the R539T-resistant isolate displayed recovery at a level nearly 3-fold greater than that seen with its revertant counterpart (Fig. 10B).

We additionally assessed whether the PB-11 *Maf1* mutant, defective in pre-tRNA regulation, displays any difference from the NF54 wild-type control with respect to recovery from artemisinin treatment. Using a 50% effective concentration (EC_50_) assay derived from the ring-stage survival assay (51), the PB-11 *Maf1* mutant parasite line displayed poorer recovery in the presence of dihydroartemisinin than the wild-type NF54 parasites (Fig. S6). Together, these results suggest there may be overlap between the pathways that regulate growth and recovery from both artemisinin treatment and amino acid starvation.

10.1128/mBio.02317-16.6 FIG S6  *Maf1* mutant parasites display decreased recovery upon dihydroartemisinin treatment. (A) Wild-type (NF54) and *Maf1* mutant (PB-11) parasites between 0 and 3 h postinvasion were treated with a range of dihydroartemisinin concentrations for a 6-h pulse and then allowed to recover for 96 h. A four-parameter log-logistic function was fitted to the relative recovery data from three biological replicates to determine the EC_50_ for recovery. NF54 EC_50_ = 0.37 nM (95% confidence interval, 0.31 nM to 0.43 nM, *P* < 0.0001); PB-11 EC_50_ = 0.25 nM (95% confidence interval, 0.23 nM to 0.27 nM, *P* < 0.00001). (B) Wild-type parasites (NF54) were capable of 50% recovery after treatment with higher doses of dihydroartemisinin (EC_50_). Error bars represent the 95% confidence intervals calculated from the log-logistic function fitted to the recovery data. Download FIG S6, TIF file, 0.5 MB. Copyright © 2017 McLean and Jacobs-Lorena. 2017 McLean and Jacobs-Lorena This content is distributed under the terms of the Creative Commons Attribution 4.0 International license.

## DISCUSSION

The *TORC1* pathway is a highly conserved signaling pathway that allows eukaryotes to modulate cellular growth in response to changes in the environment. Most of the recognizable components of this pathway have been lost in the *Plasmodium* genus. In this study, we showed that the *P. falciparum* ortholog of *Maf1* contributes to survival and recovery of parasites under conditions of various forms of growth-inhibiting stress. The observed phenotypes are consistent with those reported for other organisms, suggesting that, despite the loss of the *TORC1* pathway, *Plasmodium falciparum* encodes an analogous growth regulation pathway that acts upon this highly conserved repressor of Pol III transcription.

To study *Maf1*, we used a transposon insertion mutant produced as part of an effort to generate a genome-wide mutant collection (34). While this mutant was not a genetic knockout as previously reported, insertion of the *piggyBac* element into the *Maf1* 5′UTR resulted in profound changes in both mRNA and protein expression. This observation should serve as a reminder that the regulatory elements within transposons can affect nearby loci and that phenotypes observed after transposon insertion may be due to factors other than insertional mutagenesis alone. It remains a formal possibility that the phenotype reported here for the PB-11 mutant may have been caused in part by secondary effects on other loci not studied here. However, our observation of the mutant’s inability to regulate pre-tRNA expression and protein synthesis, phenotypes previously described for yeast, human, and *Drosophila Maf1* mutants (20, 27, 40), supports the conclusion that the mutant’s altered *Maf1* expression profile is responsible for its inability to recover from isoleucine starvation and other forms of stress.

Despite intensive efforts, we were unable to generate a knockout parasite line using standard methods, suggesting that basal *Maf1* expression may be essential for *in vitro* asexual growth. The reason that *PfMaf1* would be necessary for growth under normal conditions is not clear, as *Maf1* knockouts are viable in both yeast and mice (24, 52). One possible explanation may stem from the observation that the PB-11 *Maf1* mutant displayed elevated levels of global translation relative to the wild-type control even under normal growth conditions. *Plasmodium falciparum* schizonts are reported to globally suppress translation at the late stages of the intraerythrocytic cycle (53). It is possible that some degree of *PfMaf1* suppression of tRNA and 5S rRNA transcription is needed for the maintenance of this suppression.

In yeast and human cells, the association of *Maf1* with the RNA Pol III complex is regulated by phosphorylation. Determining whether *PfMaf1* is also phosphoregulated and identifying the kinases and phosphatases responsible for its regulation will be important next steps in understanding parasite growth regulation.

The description by Babbitt et al. of the dormancy-like slow growth response of *P. falciparum* upon isoleucine starvation was the first thorough documentation of the parasite’s ability to modulate its growth rate in response to environmental changes (12). However, after being unable to implicate any specific molecular pathways in the response (i.e., GCN2/PfIK2—IF2α), and after observing a dose-dependent correlation between the isoleucine concentration and the growth rate, they concluded that the parasite does not actively regulate its growth and that isoleucine is likely the rate-limiting factor in the progression of the cell cycle. We feel that this conclusion might have been premature. Similar conclusions were drawn in studies of other organisms, such as yeast, until it was demonstrated that chemical or genetic inhibition of *TOR* induced the same transition into stationary phase as nutrient starvation and that partial inhibition of *TOR* slowed the cell cycle in a manner similar to that seen with nutrient limitation (13, 17, 18). We believe that our description of the role of *PfMaf1* in maintaining viability upon growth retardation is the first evidence for the existence of an analogous pathway in *P. falciparum*. The remaining components of the pathway, however, have yet to be identified.

Unlike *TOR*, *Maf1* has never been shown to arrest or retard growth on its own. It is instead needed to maintain viability during the period of stress or nutrient limitation, much as we observed in *P. falciparum*. It is intriguing that the strain with the single point mutation in the *K13* gene that confers artemisinin survival also displayed increased survival upon prolonged amino acid starvation as well as greater suppression of tRNA expression. If growth is actively regulated in *Plasmodium* by a yet-to-be determined pathway, then the effects of the *K13* mutation may allow the parasite to shut down growth more effectively when faced with undesirable circumstances or to resume growth more effectively after a growth-retarding insult.

## MATERIALS AND METHODS

### Parasites.

The NF54^attB^ (used as the “wild-type” strain in this study) (54) and Dd2^attB^ (30) strains were generously provided from the laboratory of David Fidock (Columbia University). The K13 R539T artemisinin-resistant isolate (Cam3.I IPC 5202) was isolated from a patient in Battambang Province, Cambodia, in 2011. It was obtained from the Malaria Research and Reference Reagent Resource Center (MR4) for distribution by BEI Resources NIAID, NIH (product number MRA-1240), and was originally contributed by Didier Ménard (Institut Pasteur). The corresponding revertant line, Cam3.I_rev, was also obtained from BEI Resources NIAID, NIH (product number MRA-1252), and was contributed by David A. Fidock (Columbia University) (50). The PB-11 *Maf1* mutant parasite line was obtained by special request from BEI Resources NIAID, NIH (product number MRA-1031), and was contributed by John Adams (34).

### Yeast complementation.

A full-length *Plasmodium falciparum Maf1* coding sequence was synthesized with codon usage optimized for *S. cerevisiae* expression (GenScript). Using Gibson assembly (NEB; catalog no. E2611S), the core region of *PfMaf1* (amino acids 237 to 346) was fused between the *S. cerevisiae* N terminus (amino acids 1 to 233) and C-terminal “acidic tail” (amino acids 338 to 395). The resulting chimeric open reading frame was cloned into the BamHI and XhoI sites of the p416-*Met25*-3xHA yeast expression vector. The full-length yeast *Maf1* open reading frame (YDR005C) was similarly cloned into the same vector. The *Maf1*Δ knockout line (BY4741 *MAT*a library; ΔYDR005C) was transformed using the LiAc/ssDNA/PEG method (55). Survival assays were carried out on synthetic minimal dextrose medium lacking uracil (for plasmid selection) and methionine (to derepress the *Met25* promoter for transgene expression) with or without 10 nM sirolimus (Cell Signaling Technologies; catalog no. 9904).

### Parasite culture and media.

Parasites were cultured using standard methods (56) in flasks gassed with a mixture of 90% N_2_, 5% CO_2_, and 5% O_2_ (Airgas; catalog no. Z03NI9022000033). Normal culture medium consisted of RPMI 1640 with l-glutamine and 25 mM HEPES (Corning; catalog no. 10-041-CV), with 5.0 g/liter Albumax II lipid-rich bovine serum albumin (BSA; Thermo Fisher Scientific; catalog no. 11021029), 3.7 mM hypoxanthine, and 50 μg/ml gentamicin. The isoleucine-deficient medium consisted of 10.3 g/liter RPMI 1640 isoleucine (Ile) Drop-out medium (United States BioLogicals; catalog no. R9014), supplemented with 2.0 g/liter NaHCO_3_, 6.0 g/liter HEPES, 5.0 g/liter Albumax II, 3.7 mM hypoxanthine, and 50 μg/ml gentamicin. The 8 μM isoleucine medium was the same as the isoleucine medium, except that it was supplemented with 8 μM l-isoleucine. The 20% glucose media consisted of 8.4 g/liter of no-glucose RPMI 1640 (Sigma-Aldrich; catalog no. R1383), with 2.0 g/liter NaHCO_3_, 6.0 g/liter HEPES, 5.0 g/liter Albumax II, 3.7 mM hypoxanthine, 50 μg/ml gentamicin, and 0.4 g/liter glucose. Fosmidomycin was obtained from Sigma-Aldrich (catalog no. F8682-5MG), and geranylgeraniol was obtained from Santa Cruz Biotechnology (catalog no. sc-200858).

### Flow cytometry.

For each sample to be analyzed, 1 ml of parasite culture was pelleted and resuspended in 1 ml of phosphate-buffered saline (PBS) containing 4% (wt/vol) paraformaldehyde. Samples were rocked at 4°C for 20 to 30 h of fixation. Samples were then pelleted and resuspended in PBS containing 0.1% (vol/vol) Triton X-100 and rocked at room temperature for 1 h for permeabilization. This process was then repeated three additional times with PBS (without Triton X-100) to remove as much hemoglobin as possible. Samples were then diluted approximately 100-fold into normal culture medium containing 1× SYBR green I (Thermo Fisher Scientific; catalog no. S7563) and analyzed on a FACSCalibur (BD Biosciences) flow cytometer. The resulting data were further analyzed using FlowJo 10.0.7 analysis software.

### Transfection.

The *DD24*-*Maf1* construct was synthesized (GenScript) with an N-terminal 3× hemagglutinin (HA) tag followed by a *DD24* destabilization domain and a yeast codon-optimized *Maf1* open reading frame (the same synthesized construct used for yeast complementation). This open reading frame was cloned into the XhoI and XmaI sites of vector pLN-*mRPL2*pr to generate pLN-*mRPL2*pr-*DD24*-*Maf1*. This vector was used to transfect Dd2^attB^ parasites (30) by the pre-loading method (57) using a modified Bxb1 integrase transfection method (58). Stably transfected cells were maintained with 2.5 μg/ml blasticidin S. The *Shield* ligand was obtained from Aobious (catalog no. 1848).

### Genome sequencing.

High-quality, high-molecular-mass genomic DNA was prepared from 400 ml of PB-11 culture (2% hematocrit, ~10% parasitemia) enriched in late-stage parasites. Parasites were liberated from erythrocytes by saponin treatment and washed twice with PBS to remove excess hemoglobin. The resulting parasite pellet was used as starting material for genomic DNA isolation using a Blood and Cell Culture minikit (Qiagen; catalog no. 13323). Genomic DNA was sent to Axeq Technologies (Seoul, South Korea) care of the Macrogen Corporation (MD, USA) for library preparation and sequencing. An amplification-free library (59) was sequenced by paired-end sequencing to 73× coverage using an Illumina HiSeq 2000 sequencing system.

### Sequencing analysis.

The analysis of the sequencing data largely followed the protocol described by Balu et al. (35), who had previously sequenced the NF54 clone that was used as the parental line in the transposon screen which produced the PB-11 mutant (34). Two sets of paired-end reads from the parental NF54 clone (ERS038926 and ERS184445), produced by two different sequencing platforms, were obtained from the European Nucleotide Archive. These reads were used to “update” the PlasmoDB-26 *P. falciparum* 3D7 genome sequence using ICORN as part of the PAGIT software package (60). Seven iterations of ICORN generated 561 1-bp substitutions and 774 small insertions or deletions across the entire reference genome. The PB-11 reads were aligned to this updated reference sequence using bwa (61) with the default parameters. Approximately 82% of the reads aligned to the reference sequence. The resulting sam file was sorted, indexed, and filtered of duplicates using Picard-tools v 1.119. Reads were realigned around indels using the Genome Analysis Toolkit (GATK) (62), and raw variants were called using the GATK HaploTypeCaller, with ploidy set to 1. The raw variants were then filtered using vcftools v0.1.13 (63) to include only calls with a quality score greater than 60 and a minimum depth of 10 reads. The program snpEff v3 (64) was then used to filter only those variants found within open reading frames. This resulted in 32 potential variants within open reading frames. However, upon visual inspection using the Integrated Genomics Viewer v2.3.9 (65), every call was found in either a low-complexity region (e.g., an extended mononucleotide tract) or a repetitive region. In each case, there was read support for the reference sequence, leaving us to conclude that these variants more than likely represented false positives. To verify that our pipeline was indeed capable of detecting variants, we reran the pipeline analysis using the reads from the C9 *piggyBac* insertion mutant sequenced by Balu et al. (ENA ERS038913) (35). We were able to easily detect the same two SNPs reported previously.

### mRNA expression qPCR time course.

Synchronous cultures were established by sequential sorbitol treatments. For each time point, 50 ml of 2% hematocrit culture at approximately 10% parasitemia was pelleted, immediately disrupted with 10 ml of TRIzol (Thermo Fisher Scientific; catalog no. 15596018), and used for subsequent phenol-chloroform extraction and ethanol precipitation per the manufacturer’s protocol. First-strand cDNA synthesis was performed using a mixture of oligo(dT) and random hexamers and Superscript III (Thermo Fisher Scientific; catalog no. 18080044) and the standard protocol. For quantitative PCR, the *Maf1* transcript was amplified using forward primer 5′ GATGCCCACGATCGTTTTAT 3′ and reverse primer 5′ CGGAGCTAAATATTTGTGTATTGC 3′. The seryl-tRNA ligase transcript, PF3D7_0717700, was used as an internal control, with 5′ AAGTAGCAGGTCATCGTGGTT 3′ as a forward primer and 5′ TTCGGCACATTCTTCCATAA 3′ as a reverse primer. Quantitative PCR was performed using SYBR green PCR master mix (Thermo Fisher Scientific; catalog no. 4309155) on a StepOnePlus (Thermo Fisher Scientific) real-time PCR system.

### 5′-RACE.

RNA was extracted from 50 ml of a 2% hematocrit–10% parasitemia mixed-stage culture using TRIzol (Thermo Fisher Scientific; catalog no. 15596018). 5′-RACE was performed using a FirstChoice RLM-RACE kit (Thermo Fisher Scientific; catalog no. AM1700). The resulting PCR product was cloned using a CloneJET PCR cloning kit (Thermo Fisher Scientific; catalog no. K1231) and sequenced (Macrogen Corporation, USA) to determine the 5′ transcription start site.

### Recombinant protein expression and antibody production.

For recombinant protein production, the *Plasmodium berghei Maf1* ortholog (PBANKA_0718500) was selected instead of the *P. falciparum* ortholog, as it is much smaller and contains a short repeat in place of the *P. falciparum* asparagine-rich region. Outside this region, the sequences are nearly identical. The *P. berghei Maf1* sequence was synthesized with *Escherichia coli*-optimized codon usage (DNA2.0) with an N-terminal 6×His tag in a T7 promoter expression plasmid. Protein expression was conducted in NiCo21(DE3) cells (NEB; catalog no. C2529). Expression was performed using 1-liter cultures induced at an optical density at 600 nm (OD_600_) of 3.0 with 0.5 mM IPTG (isopropyl-β-d-thiogalactopyranoside)–Terrific broth for 20 h at room temperature. Cells were lysed by sonication, and protein was isolated using nickel-nitrilotriacetic acid (Ni-NTA) agarose (Qiagen; catalog no. 30210). The recombinant protein was used to produce antiserum from two separate guinea pigs by Cocalico Biologicals (PA, USA) using the standard protocol. Antiserum was affinity isolated against the recombinant *PbMaf1* protein. Western blots were visualized using a Li-COR Odyssey imaging system with 680RD linked anti-mouse (Li-COR; catalog no. 925-68070) and anti-guinea pig (Li-COR; catalog no. 925-68077) secondary antibodies. The 1-10 b *PfGAPDH* monoclonal (mouse) (66) antibody was used as a loading control.

### Growth curves.

Asynchronous parasite cultures were maintained under the conditions described and sampled every 48 h to quantify parasitemia by flow cytometry. Cultures were then diluted between 2-fold and 10-fold to maintain a parasitemia level of between 0.5% and 1.0%, and the dilution factor was recorded. The growth at each time point was measured as the parasitemia level multiplied by the dilution factor of the previous dilution. The final data set was normalized such that the initial level of parasitemia for each line was given a value of 1.0 to account for differences in the starting levels of parasitemia of the different parasite lines. A two-factor regression of the approximate hour (ln[adjusted parasitemia]) plus the genotype was fitted for each condition to test the effect of the parasite genotype on the growth rate, with time as a covariate. Doubling time (*t*_D_) was calculated for each line from single-factor regressions against time alone, using *t*_D_ = log 2/β_o_, where β_o_ is the coefficient from the single-factor regression.

### tRNA^Tyr^ intron verification and stem-loop PCR.

For tRNA analysis, RNA was isolated using a miRvana miRNA isolation kit (Thermo Fisher Scientific; catalog no. AM1560) to specifically enrich small RNAs. Due to the inherently high stability of the tRNA secondary structure, a modified reverse-transcription protocol was employed. The total volume of small RNA was mixed with a 5 μM concentration of RT primer 5′ TTACTTGTACAGCTCGTCCATGCCGAGATCCGATGAACCGGAATCG 3′, which anneals to the 3′ end of the tRNA^Tyr^ (PF3D7_0702800) sequence but contains a RACE-like 5′ extension of an unrelated primer sequence for subsequent PCR amplification (to avoid amplifying the genomic locus). The small-RNA–primer mixture was heated to 98°C for 5 min and then cooled slowly to 4°C. Reverse transcription was then performed using Superscript III (Thermo Fisher Scientific; catalog no. 18080044) and the manufacturer’s protocol, with the exception that it was performed at the elevated temperature of 65°C for 1 h to minimize the effects of the presence of the tRNA secondary structure. The cDNA was then amplified using Phusion High-Fidelity polymerase (NEB; catalog no. M0530L), forward primer 5′ CCGATGATAGCTCAGTTGGTAGA 3′ (which anneals to the 5′ end of the tRNA^Tyr^ sequence), and reverse primer 5′ CTTGTACAGCTCGTCCATGCC 3′ (which anneals to the 5′ extension of the RT primer). The resulting PCR product was cloned using a CloneJET PCR Cloning kit (Thermo Fisher Scientific; catalog no. K1231) and sequenced (Macrogen Corporation) to verify the excision of the predicted 11-nt intron.

### Pre-tRNA^Tyr^ stem-loop qPCR.

For analysis of pre-tRNA^Tyr^, highly synchronous parasite cultures were initiated by Percoll-sorbitol treatment to generate 50 ml of 2% hematocrit culture with a level of parasitemia of between 5% and 10% and with a maximum parasite age of 4 hpi. These cultures were then washed three times in 50 ml PBS and resuspended in either normal media or media lacking isoleucine and then incubated for 24 h prior to harvesting. Cultures were pelleted, treated with 0.1% saponin–PBS, and washed twice with 50 ml PBS to remove liberated hemoglobin. A small RNA sample was then isolated from parasite pellets using a miRvana miRNA isolation kit (Thermo Fisher Scientific; catalog no. AM1560) according to the manufacturer’s protocol. In order to specifically detect the presence of the 11-nt intron in the pre-tRNA^Tyr^, a stem-loop PCR method originally developed for miRNA detection (45, 46) was adapted. In this method, a self-annealing 5′ stem-loop extension to the RT primer adds length to the final product for downstream qPCR amplification product and may also provide additional stability for the annealing of the short complementary primer sequence due to stacking interactions provided by the stem-loop. For reverse transcription, small RNA samples were combined with a 5* *μM mixture of the stem-loop RT primers 5′ GTCGTATCCAGTGCAGGGTCCGAGGTATTCGCACTGGATACGACATAACCATTTC 3′ and GTCGTATCCAGTGCAGGGTCCGAGGTATTCGCACTGGATACGACCAGCATTCTGA, which anneal to the pre-tRNA^Tyr^ intron and the 3′ end of 5.8S rRNA, respectively. The primer-RNA mixture was heated to 98°C for 5 min and then cooled to 4°C. Reverse transcription was carried out at 65°C for 1 h to process the RNA secondary structure correctly but otherwise followed the Superscript III protocol of the manufacturer (Thermo Fisher Scientific; catalog no. 18080044). Quantitative PCR was performed using the common reverse primer 5′ CAGTGCAGGGTCCGAGGT 3′ (which anneals to the shared stem-loop structure) and the gene-specific forward primers 5′ AGTTGGTAGAGCGGCAGACT 3′ (pre-tRNA^Tyr^) and 5′ AGCAAAACGCGATAAGCAAT 3′ (5.8S rRNA) on a StepOnePlus real-time PCR system (Thermo Fisher Scientific) and Power SYBR green master mix (Thermo Fisher Scientific; catalog no. 4367659).

### Puromycylation assay.

Cultures were initiated using Percoll-sorbitol synchronization to produce 50-ml cultures at 2% hematocrit and approximately 10% parasitemia. Cultures were then pelleted and washed three times with 50 ml PBS and resuspended in either normal or isoleucine-lacking medium. After 24 h, puromycin was added to reach a final concentration of 1.0* *μM and parasites were incubated for an additional 1 h. Samples were then pelleted and resuspended in 50 ml 0.1% saponin–PBS and were subsequently washed twice with 50 ml PBS to remove hemoglobin. Parasite pellets were then lysed using radioimmunoprecipitation assay (RIPA) buffer containing protease inhibitors. For ELISA, 500 ng/well of each biological replicate was separately probed with either the anti-puromycin monoclonal antibody 3RH11 (Kerafast; catalog no. EQ0001) or monoclonal anti-*Plasmodium falciparum GAPDH* as an internal control. As a negative control, parasites grown in normal media were treated with 10 μg/ml cycloheximide simulataneously with puromycin to arrest protein synthesis and block puromycin incorporation.

### Ring-stage-survival EC_50_ assay.

Schizonts were isolated from Percoll gradients and allowed to invade fresh erythrocytes for 3 h, after which a sorbitol synchronization was performed. Synchronous 0-to-3-h-old rings were immediately divided into 1-ml (2% hematocrit) volumes of replicate wells and treated with 12 steps of dihydroartemisinin diluted 3-fold in dimethyl sulfoxide (DMSO) beginning with a maximum concentration of 10 μM. After 6 h of drug exposure, the contents of each 1-ml well of culture were pelleted and washed with 15 ml of fresh medium to remove drug and then returned to a 1-ml culture for an additional 90 h of recovery. At the end of the 96-h assay, cultures were fixed and quantified by flow cytometry as described above. EC_50_ values were calculated using a four-parameter log-logistic function and the *drc* package in R (67).

### Statistical analysis.

All statistical analyses were performed using R version 3.2.3 (10 December 2015) (68). *t* tests, linear and logistic regressions, and some figures were generated using R base functions. The *drc* package (67) was used for determining the 50% survival point of PB-11, and the *ggplot2* package (69) was used for the production of several figures.

### Accession number(s).

The paired-end read data determined in this work have been deposited in the NIH Sequence Read Archive under accession number SRR4206195.
