# Supplementary figures and images for: Plasmodium falciparum Maf1 Confers Survival upon Amino Acid Starvation
Source: mBio. 2017 Mar 28;8(2):e02317-16. doi: 10.1128/mBio.02317-16 (PMC5371417; doi:10.1128/mBio.02317-16)

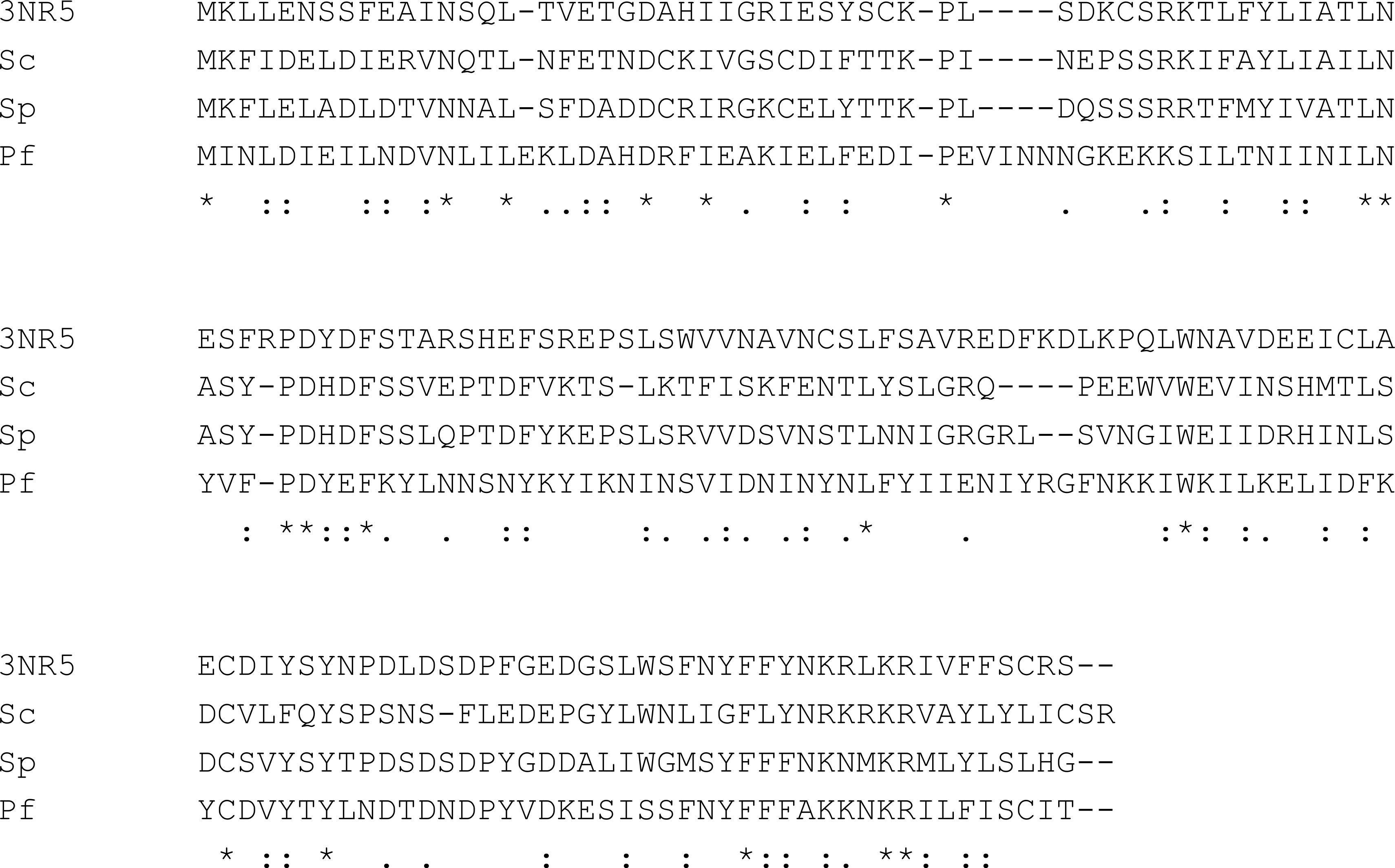

Supplement: FIG S1 [file mbo002173248sf1.tif]

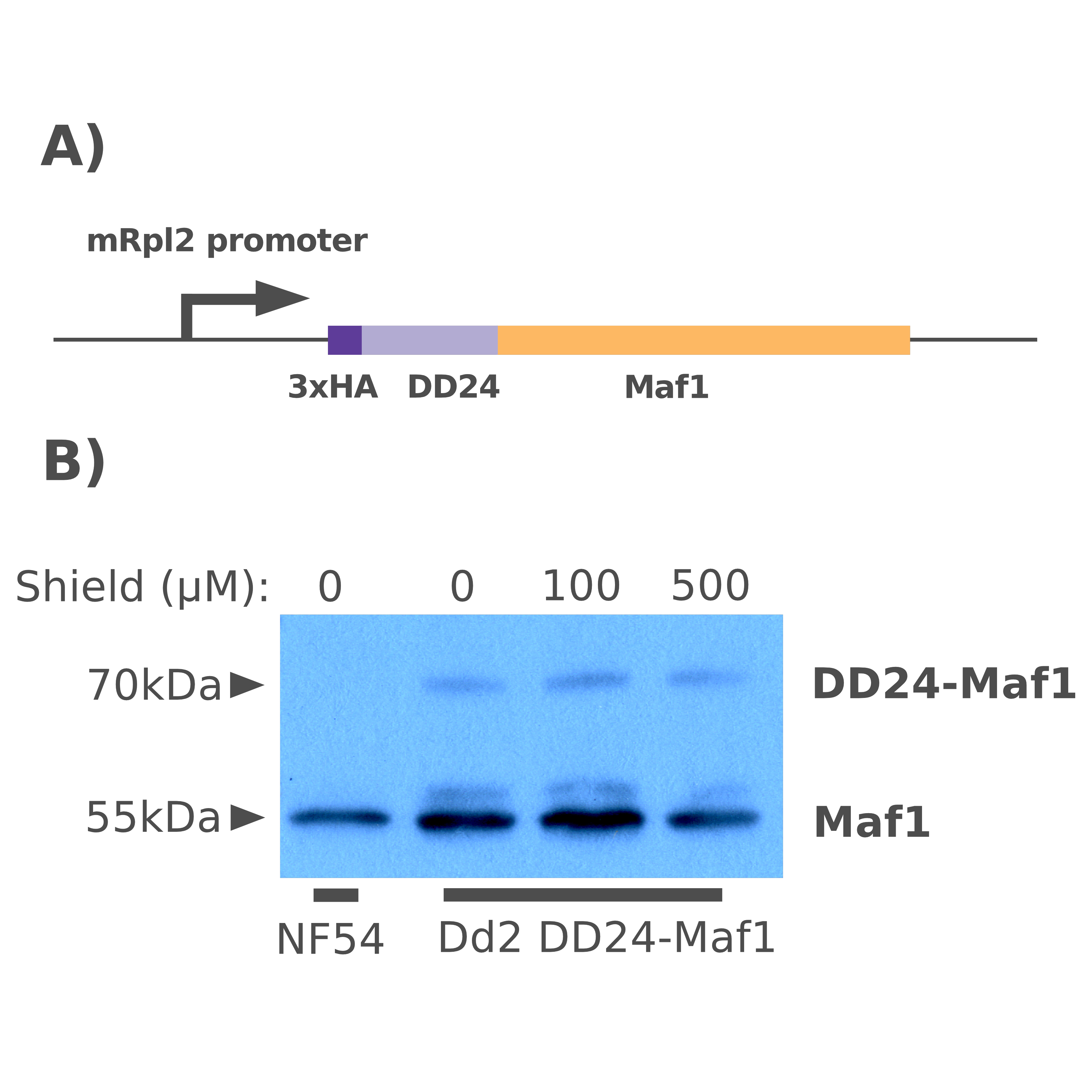

Supplement: FIG S2 [file mbo002173248sf2.tif]

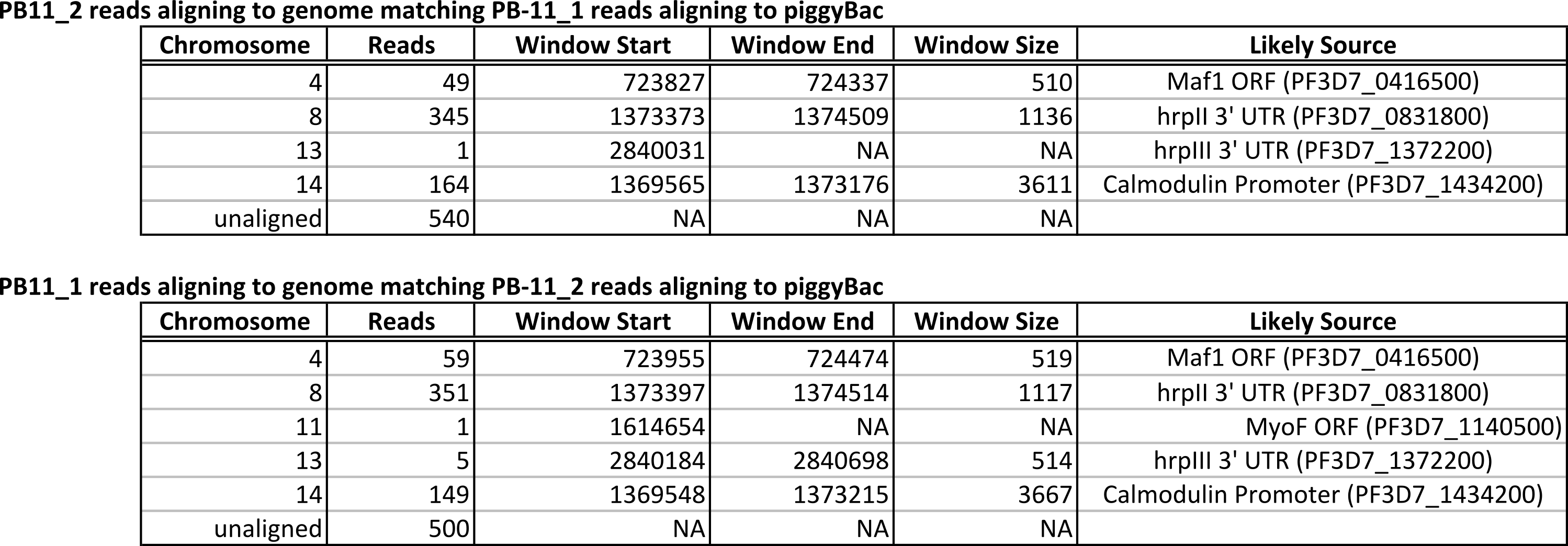

Supplement: TABLE S1 [file mbo002173248st1.tif]

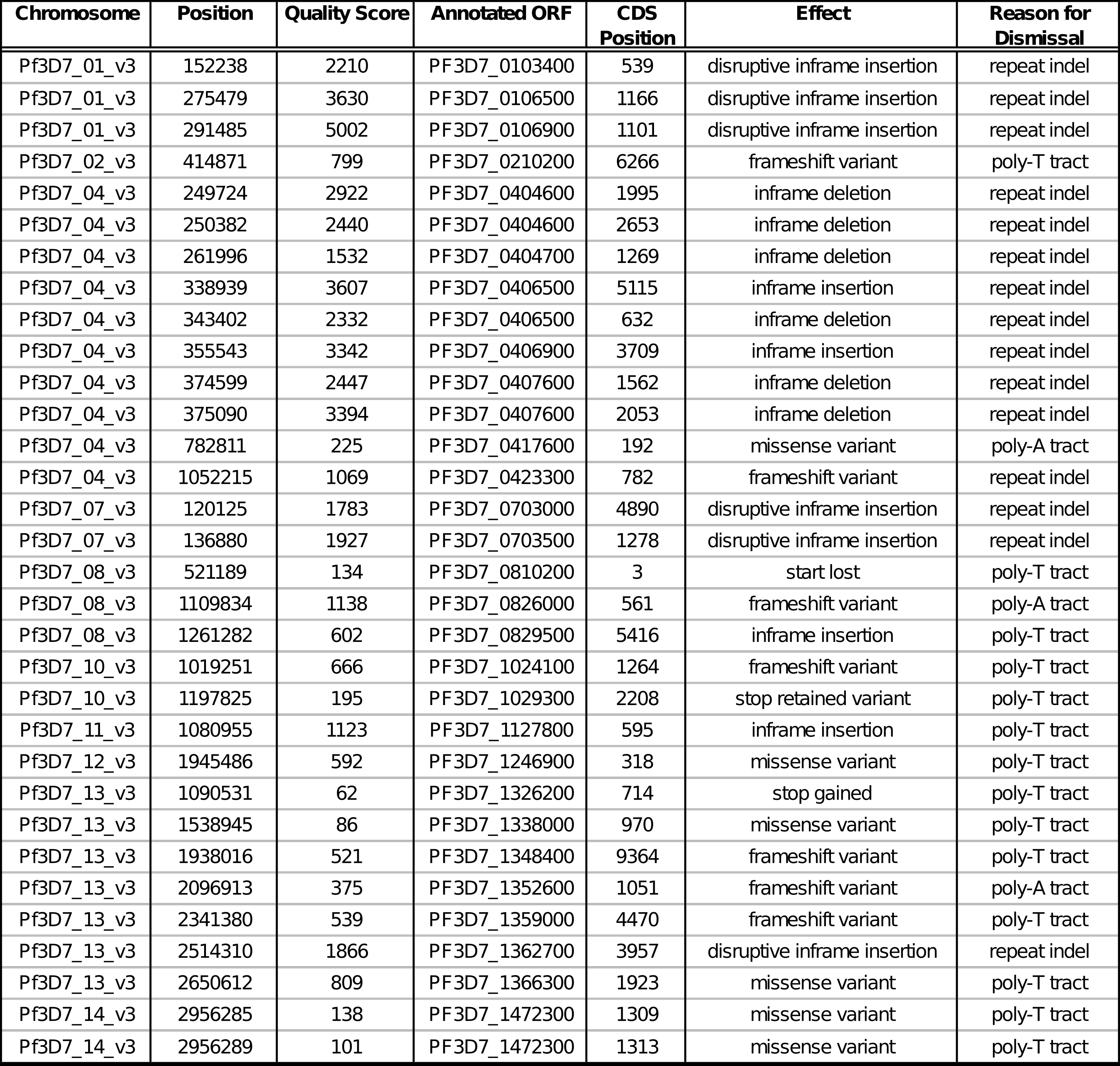

Supplement: TABLE S2 [file mbo002173248st2.tif]

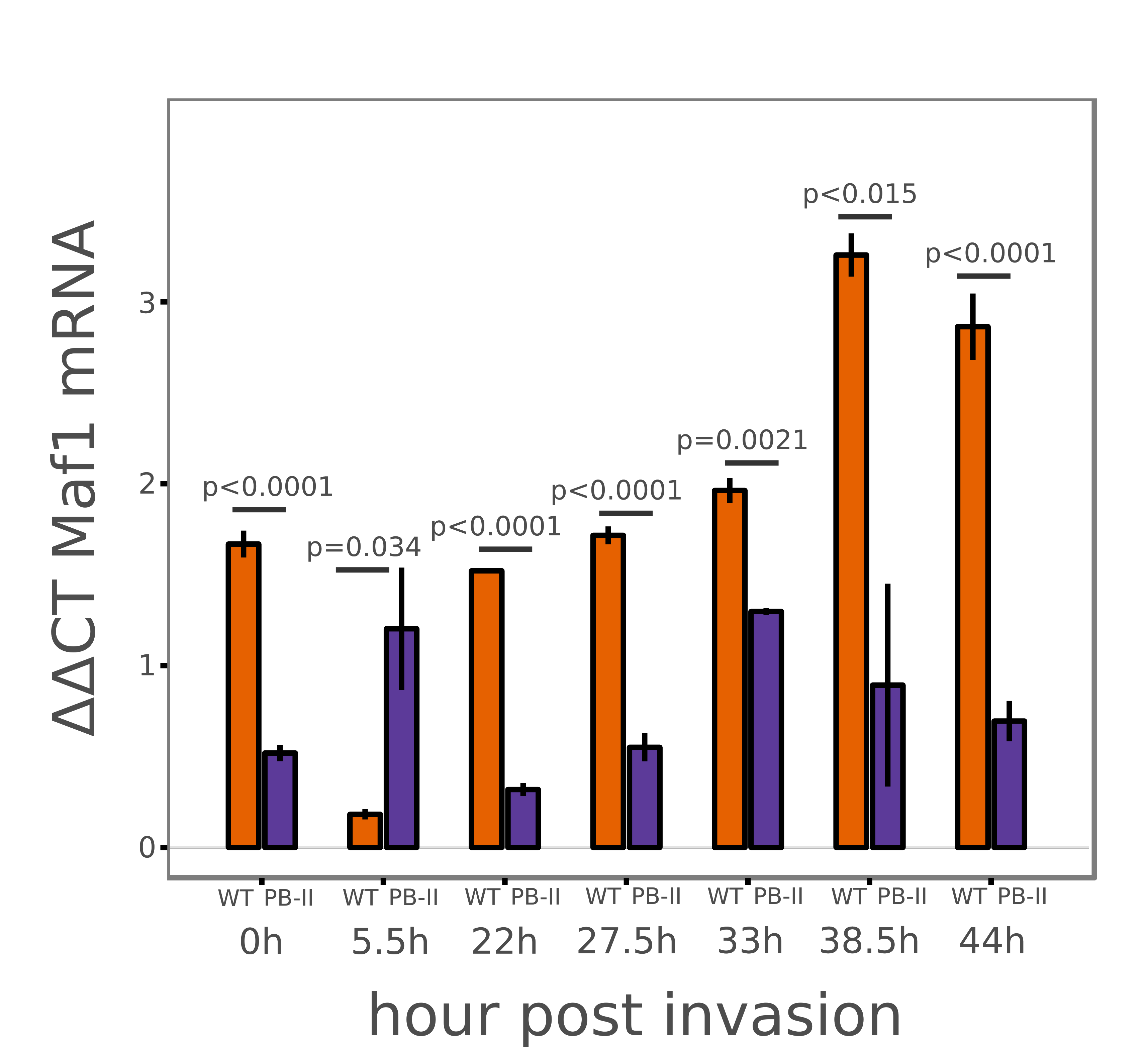

Supplement: FIG S3 [file mbo002173248sf3.tif]

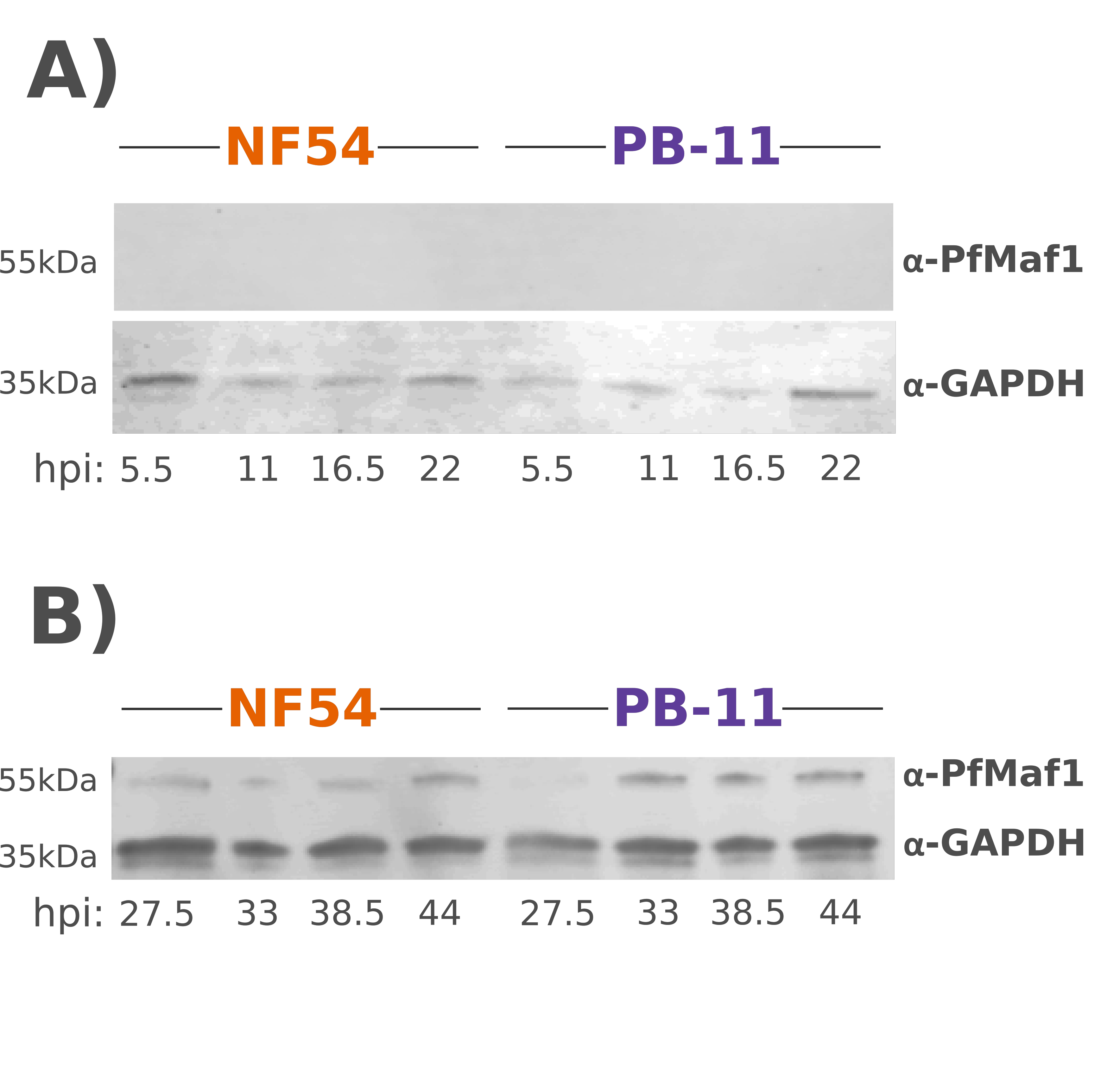

Supplement: FIG S4 [file mbo002173248sf4.tif]

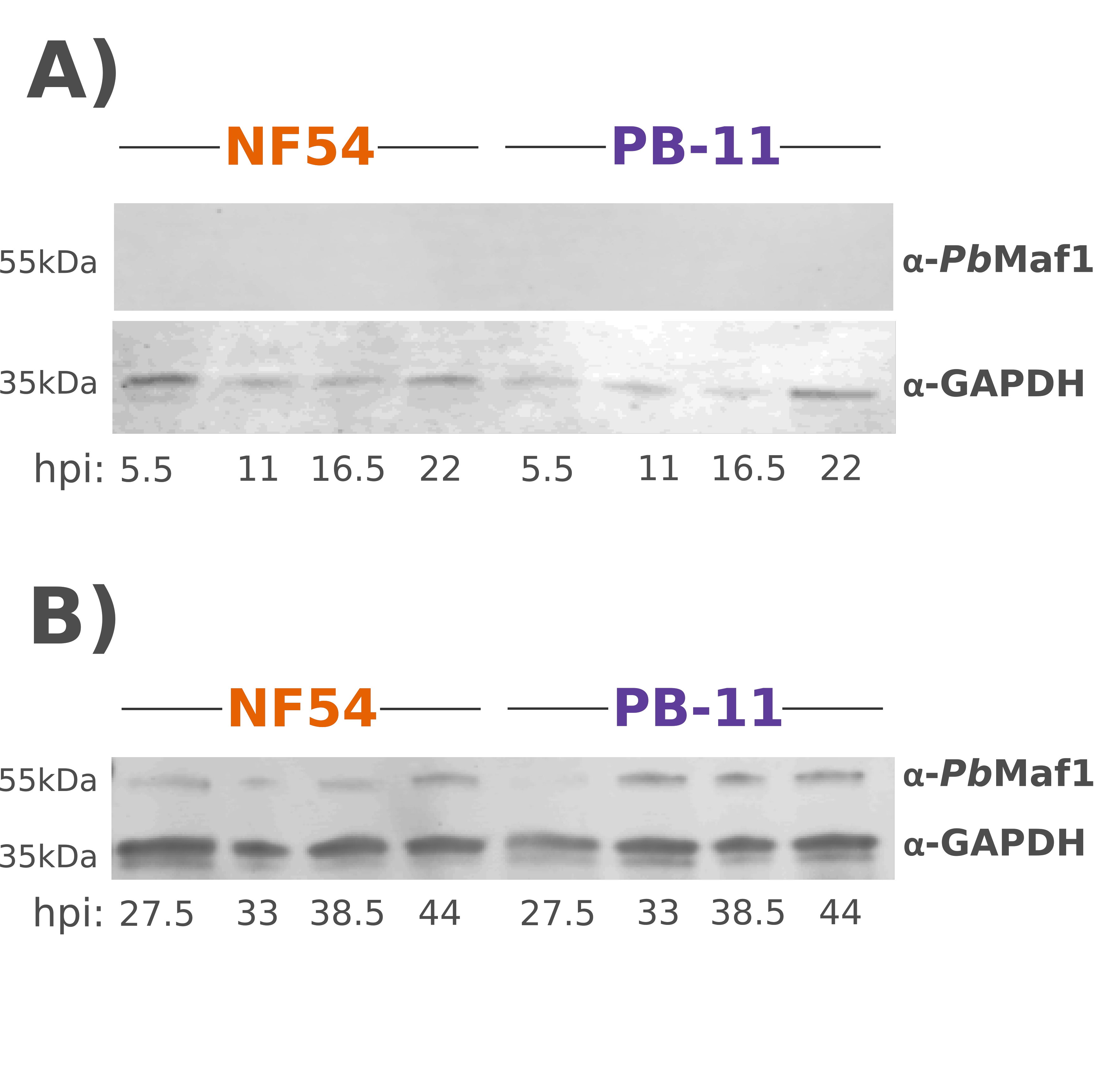

Supplement: FIG S5 [file mbo002173248sf5.tif]

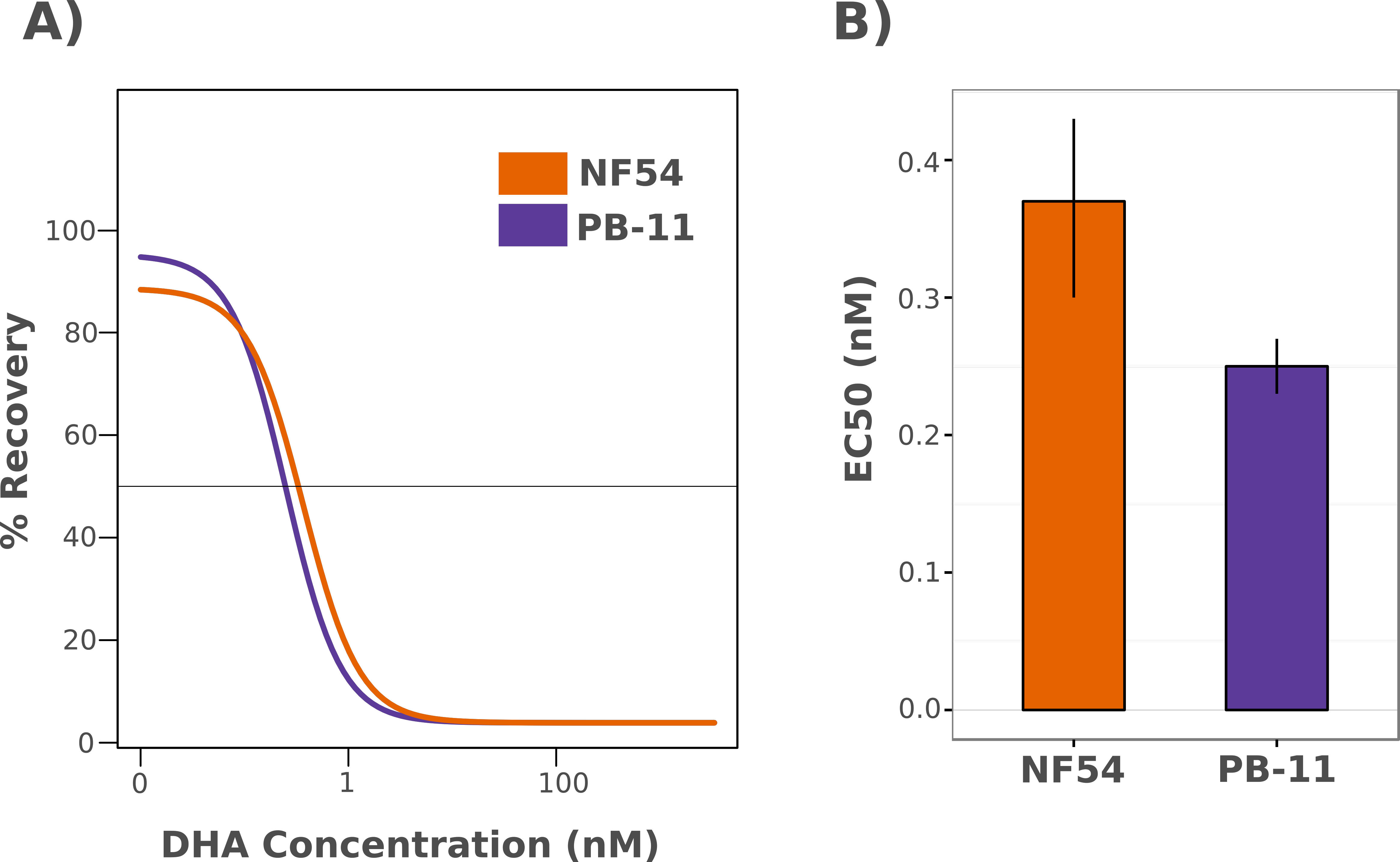

Supplement: FIG S6 [file mbo002173248sf6.tif]
